# SIRT7 orchestrates melanoma progression by simultaneously promoting cell survival and immune evasion via UPR activation

**DOI:** 10.1038/s41392-023-01314-w

**Published:** 2023-03-15

**Authors:** Xiuli Yi, Huina Wang, Yuqi Yang, Hao Wang, Hengxiang Zhang, Sen Guo, Jianru Chen, Juan Du, Yangzi Tian, Jingjing Ma, Baolu Zhang, Lili Wu, Qiong Shi, Tianwen Gao, Weinan Guo, Chunying Li

**Affiliations:** Department of Dermatology, Xijing Hospital, Fourth Military Medical University, No 127 of West Changle Road, Xi’an, Shaanxi 710032 China

**Keywords:** Skin cancer, Cancer microenvironment

## Abstract

Melanoma is the most lethal type of skin cancer, originating from the malignant transformation of melanocyte. While the development of targeted therapy and immunotherapy has gained revolutionary advances in potentiating the therapeutic effect, the prognosis of patients with melanoma is still suboptimal. During tumor progression, melanoma frequently encounters stress from both endogenous and exogenous sources in tumor microenvironment. SIRT7 is a nuclear-localized deacetylase of which the activity is highly dependent on intracellular nicotinamide adenine dinucleotide (NAD^+^), with versatile biological functions in maintaining cell homeostasis. Nevertheless, whether SIRT7 regulates tumor cell biology and tumor immunology in melanoma under stressful tumor microenvironment remains elusive. Herein, we reported that SIRT7 orchestrates melanoma progression by simultaneously promoting tumor cell survival and immune evasion via the activation of unfolded protein response. We first identified that SIRT7 expression was the most significantly increased one in sirtuins family upon stress. Then, we proved that the deficiency of SIRT7 potentiated tumor cell death under stress in vitro and suppressed melanoma growth in vivo. Mechanistically, SIRT7 selectively activated the IRE1α-XBP1 axis to potentiate the pro-survival ERK signal pathway and the secretion of tumor-promoting cytokines. SIRT7 directly de-acetylated SMAD4 to antagonize the TGF-β-SMAD4 signal, which relieved the transcriptional repression on IRE1α and induced the activation of the IRE1α-XBP1 axis. Moreover, SIRT7 up-regulation eradicated anti-tumor immunity by promoting PD-L1 expression via the IRE1α-XBP1 axis. Additionally, the synergized therapeutic effect of SIRT7 suppression and anti-PD-1 immune checkpoint blockade was also investigated. Taken together, SIRT7 can be employed as a promising target to restrain tumor growth and increase the effect of melanoma immunotherapy.

## Introduction

Melanoma is the most lethal type of skin cancer, resulting from the malignant transformation of melanocytes, with its incidence continuously increasing worldwide and pathogenesis of considerable complexity.^1^ Although targeted therapy and immunotherapy have substantially potentiated the therapeutic effect, the inevitable development of treatment resistance and low response rate significantly hinder the therapeutic efficacy of melanoma treatment.^2,3^ Therefore, it is of rather necessity to further study the mechanism underlying melanoma pathogenesis and develop alternative therapeutic paradigm to optimize the outcome of patients with melanoma.

The tumor microenvironment (TME) of solid tumor is comprised of distinct cell populations in a complex matrix, which often exhibits insufficient nutrient supply, inadequate oxygen delivery, ineffective waste removal and poorly-differentiated vasculature that result in hypoxia, acidosis, and nutrients deprivation.^4^ Thus facing such various stress, tumor cells activate intrinsic protective signals for survival to prevent from succumbing to apoptosis.^5,6^ Sirtuins are a family of deacetylases of which the activity are highly dependent on intracellular NAD^+^ level. They can sense nutrients and regulate cell homeostasis, in particular exert a cyto-protective effect in response to various exogenous environmental stresses.^7–9^ Mammalian genome encodes seven members of sirtuins (SIRT1~SIRT7), among which SIRT7 is exclusively localized in nuclear that has been initially demonstrated to link H3K18 de-acetylation to oncogenic transformation.^10^ Accumulative evidence has revealed that SIRT7 manipulates ribosomal biogenesis, glucose homeostasis, mitochondrial biogenesis and genotoxic stress response,^11–13^ indicating its indispensable role in maintaining cellular homeostasis and adapting to exogenous stress. More importantly, the dysregulated expression or activity of SIRT7 was pivotal in cancer biology, whereas its actual role is tumor type-specific and context-dependent. For one thing, SIRT7 plays pro-tumorigenic roles in prostate cancer, glioma, breast cancer and thyroid cancer via the regulation of autophagy, ERK/STAT3 signal, p38 MAPK and Akt-p70S6K1 axis respectively.^14–17^ For another, the notion that SIRT7 acts as a tumor suppressor is supported by the findings in oral squamous cell carcinoma and colon cancer via its de-acetylation activity of SMAD family member 4 (SMAD4) and WD repeat domain 77 (WDR77).^18,19^ In addition to regulating tumor cell malignant behavior, tumorous SIRT7 expression is highly associated with M1 macrophages infiltration and T cell exhaustion in breast cancer-luminal, and suppressed the recruitment and activation of T cells in hepatocellular carcinoma cells, respectively.^20,21^ Therefore, the disruption of SIRT7 can prominently promote the effect of immunotherapy in hepatocellular carcinoma,^20^ indicating that tumorous SIRT7 can shape TME and modulate anti-tumor immunity as well. Nevertheless, whether SIRT7 participates in regulating tumor cell biology and anti-tumor immunity in melanoma, especially under the circumstance of stressful TME, has not been investigated.

Cytotoxic conditions of TME usually trigger endoplasmic reticulum (ER) stress, which induces the activation of unfolded protein response (UPR) to cope with stress and help to normalize ER function.^22^ UPR process is initiated by the following three independent ER trans-membrane proteins, namely, activating transcription factor 6 (ATF6), RNA-dependent protein kinase-like ER kinase (PERK), and inositol-requiring enzyme 1α (IRE1α/ERN1). To be specific, the activation of endoribonuclease activity of IRE1α promotes the splicing of X-box binding protein 1 (XBP1) mRNA, which then transcriptionally induces the expressions of molecules responsible for normalizing protein folding and maintaining cell homeostasis.^23^ Furthermore, three main branches of the mitogen activated kinase-like protein (MAPK) pathway, extracellular regulated protein kinases (ERK), c-Jun NH2-terminal kinase (JNK), and p38 MAPK, can also be activated by IRE1α under ER stress through various mechanisms to link UPR signal to cell survival.^24,25^ Of note, tumorous IRE1α-XBP1 axis activation also reshapes the tumor microenvironment, not only by inducing the secretion of tumor-promoting cytokines like Interleukin-8 (IL-8), tumor necrosis factor α (TNF-α), and vascular endothelial growth factor (VEGF) to modulate immune cell function and angiogenesis,^26^ but also by regulating immune checkpoint or intrinsic signaling pathway to eradicate or potentiate anti-cancer immunosurveillance.^27^ Therefore, IRE1α-XBP1 is a central regulatory axis orchestrating tumor progression under ER stress. A series of previous reports has elucidated the upstream mechanisms responsible for the activation of IRE1α-XBP1 and other UPR signal branches in tumor and other diseases, including mutated oncogenic drivers *BRAF*, *c-myc*^28,29^ and post-translational regulations.^30–33^ Nevertheless, the molecular linkage between the nutrient-responsive sirtuins family and the IRE1α-XBP1 axis of UPR in stressful TME and the implication in melanoma tumor biology remains elusive.

Herein, we first identified that in the sirtuins family, SIRT7 expression was the most significantly increased one upon nutrient deprivation and ER stress inducer treatment that mimic stressful TME. Then, we proved that the knockdown of SIRT7 exerted marginal effects on melanoma cell proliferation under normal conditions, whereas it potentiated tumor cell death under ER stress. In parallel, SIRT7 expression deficiency significantly suppressed melanoma growth in vivo by promoting tumor cell apoptosis. Mechanistically, SIRT7 selectively activated the IRE1α-XBP1 axis to potentiate the pro-survival ERK signal pathway and the secretion of tumor-promoting cytokines like IL-8, TNF-α and VEGFA. To be specific, SIRT7 directly de-acetylated SMAD4 to antagonize the TGF-β-SMAD4 signal, which relieved the transcriptional repression on IRE1α, so that resulted in increased activation of the IRE1α-XBP1 axis. Aside from direct regulation of tumor cell behavior, the results from xenograft tumor in immune-competent mice showed increased CD8^+^T cells infiltration in TME in the tumor with SIRT7 deficiency. The suppressive effect of SIRT7 deficiency on melanoma progression was CD8^+^T cell-dependent, which was partially caused by the reduction of PD-L1 via the IRE1α-XBP1 axis. Ultimately, the synergized therapeutic effect of SIRT7 suppression and immune checkpoint blockade was also investigated.

## Results

### SIRT7 is a stress-responsive factor and significantly up-regulated in melanoma

**Fig. 1:**
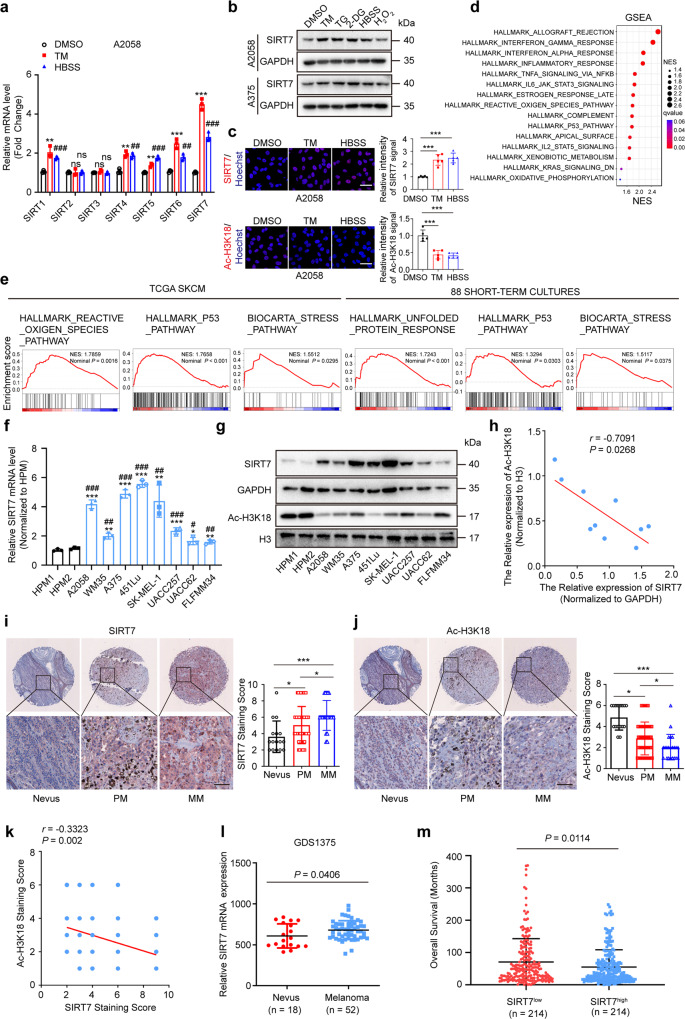
SIRT7 is a stress-responsive factor and significantly up-regulated in melanoma. **a** Relative mRNA fold change of all sirtuins family members in A2058 cells treated with TM (3 μM) or HBSS for 24 h. **b** Immunoblotting analysis of SIRT7 expression in A2058 and A375 cells treated with TM (3 μM), TG (1 μM), 2-DG (10 mM), HBSS or H_2_O_2_ (400 μM) for 24 h. **c** Immunofluorescence staining of SIRT7 and Ac-H3K18 in A2058 cells treated with TM (3 μM) or HBSS for 24 h. Scale bar, 50 μm. **d** Representative pathways associated with SIRT7 expression in TCGA SKCM database. **e** GSEA analysis between SIRT7 expression and reactive oxygen species, unfolded protein response, P53 pathway and BIOCARTA stress pathway in TCGA SKCM database and 88 melanoma cell lines short-term cultures database. **f** Relative mRNA level of *SIRT7* in melanoma cell lines and two cultures of HPMs. **g**, **h** Immunoblotting analysis of SIRT7 expression and quantified graph in melanoma cell lines and two cultures of HPMs. **i**, **j** Immunohistochemical staining analysis of SIRT7 and Ac-H3K18 in TMA. Scale bar, 100 μm. **k** Correlation analysis of SIRT7 and Ac-H3K18 scores in TMA. **l** SIRT7 expression in melanoma and nevus in Talantov dataset (GDS1375). **m** The comparison of the survival between patients with low SIRT7 level and high SIRT7 level in TCGA SKCM database. Data represent the mean ± SD of triplicates. *r* value was calculated by Spearman correlation*. P* value was calculated by two tailed Student’s *t*-test, *P < 0.05, **P < 0.01, ***P < 0.001, HPM#1 group as control; ^#^*P* < 0.05, ^##^*P* < 0.01, ^###^*P* < 0.001, HPM#2 group as control. ns non-significant

Solid tumors including melanoma commonly encounter stress from nutrient deprivation, hypoxia, and other cytotoxic stimuli in TME.^34^ To mimic this status in vitro, melanoma cells were stimulated by 2-deoxy-D-glucose (2-DG), Hanks’ balanced salt solution (HBSS), Hydrogen Peroxide (H_2_O_2_), or ER stress inducer Tunicamycin (TM) or Thapsigargin (TG) that resembled glucose/nutrient shortage or extrinsic stress,^35–38^ which all resulted in prominent up-regulation of HSPA5 (ER stress indicator) and P53 (stress-associated factor), indicating that tumor cells are undergoing stress (Supplementary Fig. S1a). Of note, these treatments induced prominent alteration of cellular energetic status, revealed by the decline of NADH level and increase of NAD^+^ level, and the consistent increase of the ratio of NAD^+^ to NADH (Supplementary Fig. S1b), indicating the impairment of intracellular energy homeostasis. Considering sirtuins are NAD^+^-dependent deacetylases whose function could be activated upon NAD^+^ level increase, we queried the alterations of the sirtuins family under stressful circumstances. Through qRT-PCR analysis of all sirtuins family members in A2058 melanoma cells treated with TM or HBSS, it was found that SIRT7 displayed the most prominent up-regulation than other members of sirtuins (Fig. 1a). Immunoblotting assay confirmed the robust increase of SIRT7 protein level after the treatment with 2-DG, HBSS, H_2_O_2_, TM or TG (Fig. 1b). Besides, immunofluorescences staining analysis also revealed the induction of SIRT7 after the treatment with TM or HBSS (Fig. 1c). Given that SIRT7 is a promoter-associated, highly selective H3K18 deacetylase,^10^ we detected ac-H3K18 level to reveal the deacetylase activity of SIRT7. Parallel to the up-regulation of SIRT7 and the increase of NAD^+^ abundance, the level of ac-H3K18 significantly declined after the treatment with TM or HBSS (Fig. 1c), suggesting the potentiation of SIRT7 enzymatic activity upon stress. For further verification, we turned to TCGA skin cutaneous melanoma (SKCM) database to analyze the relationship between SIRT7 expression and tumor stress status. All 473 available cases were divided into two groups as SIRT7^high^ and SIRT7^low^ according to the median value of SIRT7, and gene set enrichment analysis (GSEA) was employed to find the gene expression signatures that correlated with SIRT7 expression. Among the top 15 enriched hallmark pathways, inflammatory responses, reactive oxygen species (ROS), and P53 pathway that were highly correlated with stress were enlisted (Fig. 1d, e). SIRT7 expression was also associated with BIOCARTA stress pathway (Fig. 1e). Similar to the alteration in response to 2-DG, HBSS, H_2_O_2_, TM, or TG, both the mRNA and protein levels of SIRT7 were prominently increased under hypoxic conditions (Supplementary Fig. S1c). Additionally, GSEA analysis in 88 melanoma cell lines short-term cultures also revealed that SIRT7 expression was strongly correlated with unfolded protein response and P53 pathway (Fig. 1e). Besides, we also referred to the Gene Expression Omnibus (GEO) melanoma dataset (GSE19234) to analyze the relationship between SIRT7 expression and tumor stress status. Congruently, SIRT7 expression was prominently correlated with Reactive oxygen species (ROS) pathway, P53 pathway, and BIOCARTA stress pathway (Supplementary Fig. S1d). Taken together, SIRT7 is generally a stress-responsive factor of which the expression and activity can be induced upon stress caused by nutrient deprivation or pharmacological stimulation.

Thereafter, we also examined the expression status of SIRT7 under normal conditions in melanoma. Our analysis in a panel of melanoma cell lines and human primary melanocyte (HPM) revealed that both the mRNA and protein levels of SIRT7 were significantly increased in melanoma cells compared to HPM (Fig. 1f, g), which might be related to higher basal ER stress in melanoma than that in the melanocyte.^28^ Meanwhile, the level of ac-H3K18 was consistently reduced in melanoma cells (Fig. 1g), and there is a prominent negative correlation between SIRT7 level and ac-H3K18 level in melanoma cell lines and melanocytes (Fig. 1h). We subsequently analyze the genomic profile of SIRT7. Among 470 samples with both available DNA and RNA sequencing data, SIRT7 genetic alteration occurred in 14% of total cases, and the majority displayed an increase in SIRT7 expression (amplification and mRNA up-regulation) (Supplementary Fig. S1e). SIRT7 copy number was significantly correlated with its mRNA expression (Supplementary Fig. S1f), and the up-regulation of SIRT7 seemed to partially result from its ploidy status (gain and amplification) (Supplementary Fig. S1g) since its copy number revealed a similar pattern as the amplified oncogene MITF (Supplementary Fig. S1h). For further verification, immunohistochemical staining analysis of SIRT7 was performed via the employment of a tumor tissue microarray (TMA) containing 18 melanocytic nevus tissues, 62 primary melanoma tissues, and 20 metastatic melanoma tissues. SIRT7 staining score was significantly higher in melanomas than nevus, and revealed more prominent increase in metastatic melanomas compared to primary ones (Fig. 1i). In parallel, the staining score of ac-H3K18 showed a significant reduction in melanomas compared to nevus, which was more prominent in metastatic melanomas that in primary ones (Fig. 1j). SIRT7 staining score was in prominent negative correlation with ac-H3K18 (Fig. 1k). Moreover, SIRT7 expression was significantly higher in melanoma than in nevus in Talantov dataset (GDS1375) (Fig. 1l). Although cases in TCGA SKCM database categorized into two groups by median SIRT7 level showed no significance of overall survival through Kaplan-Meier survival analysis (data not shown), cases with low SIRT7 mRNA level tended to have longer average overall survival than those with high SIRT7 mRNA level (Fig. 1m). These results provide in vivo evidence that SIRT7 was significantly increased in melanoma and was highly correlated with stress-related indicators.

### SIRT7 enables melanoma cell survival under stress

**Fig. 2:**
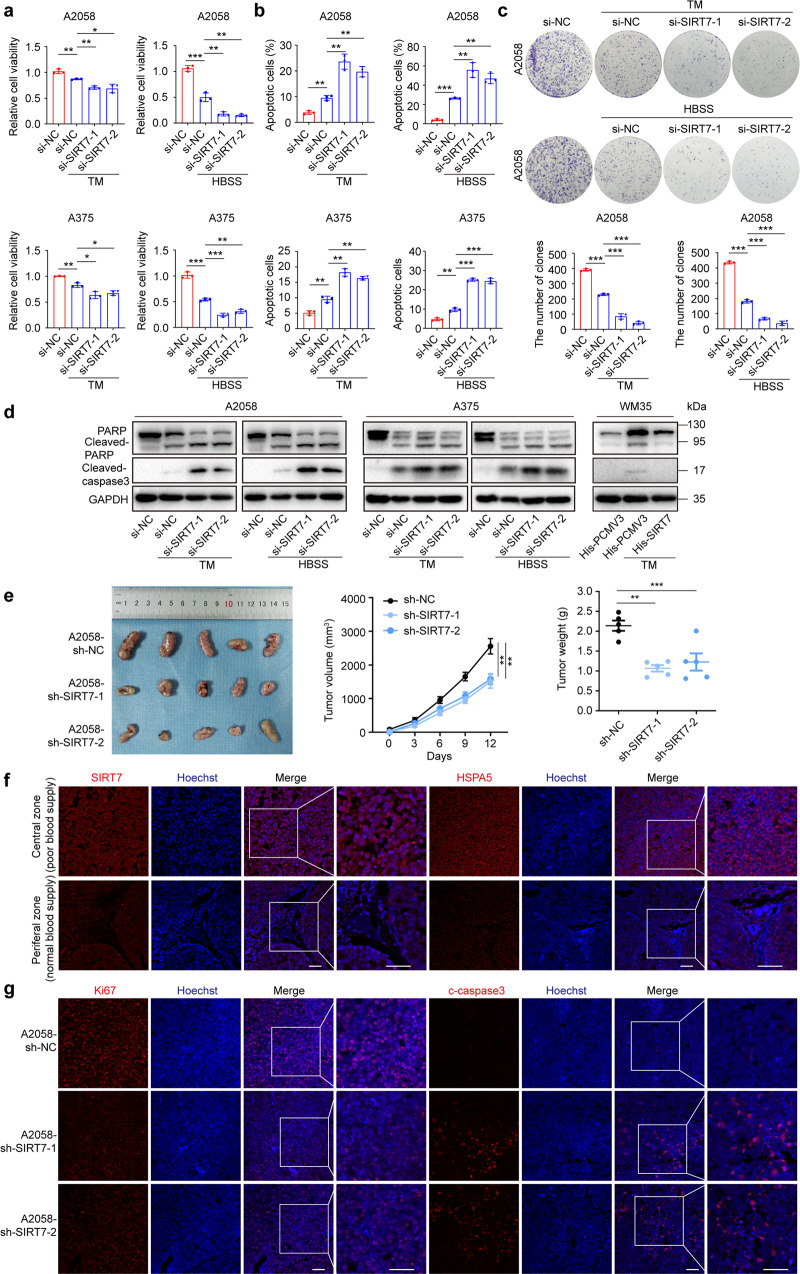
SIRT7 enables melanoma cell survival under stress. **a**, **b** Cell viability and apoptosis rate of A2058 and A375 melanoma cells treated with TM (3 μM) or HBSS for 24 h with or without the knockdown of SIRT7. **c** Colony formation of A2058 cells treated with TM (3 μM) or HBSS for 24 h with or without the knockdown of SIRT7. **d** Immunoblotting analysis of apoptosis-related molecules expression in A2058, A375 and WM35 cells with indicated treatment. **e** Images of A2058 xenografts with indicated treatment isolated from nude mice. Tumor volumes and weights in each group were calculated and displayed on the right. Symbols of one dot indicates one mouse, and the error bars are mean with ±S.D. **f** Immunofluorescence staining of SIRT7 and HSPA5 in tumors from nude mice. Scale bar, 50 μm. **g** Immunofluorescence staining of ki67 and cleaved-caspase3 in tumors from nude mice. Scale bar, 50 μm. Data represent the mean ± SD of triplicates. *P*-value was calculated by two-tailed Student’s *t*-test. *P < 0.05, **P < 0.01, ***P < 0.001

Thereafter, we went on to investigate the actual role of SIRT7 in melanoma. Two independent siRNA against *SIRT7* were employed to knockdown SIRT7 to avoid the potential off-target effect (Supplementary Fig. S2a). Unexpectedly, the knockdown of SIRT7 exerted a marginal effect on either cell viability in a short term or cell proliferation in a long term as revealed by CCK8 and colony formation assay, respectively (Supplementary Fig. S2b), indicating that SIRT7 was dispensable for melanoma cell proliferation under normal condition. Regarding SIRT7 being a stress-responsive factor according to our results, we turned to figure out whether SIRT7 plays a regulatory role in melanoma cells upon exogenous stress. Melanoma cells were pre-transfected with siRNA against *SIRT7* and then processed to the treatment with TM (ER stress inducer) or HBSS (nutrient deprivation). As was shown by the CCK8 assay, SIRT7 deficiency forwardly impaired short-term cell viability caused by these two stress inducers in both cell lines (Fig. 2a). In parallel, flow cytometry analysis displayed that SIRT7 deficiency exacerbated cell death in the face of these stimuli (Fig. 2b; Supplementary Fig. S2c), whereas the overexpression of SIRT7 could suppress cell death caused by the treatment with TM in WM35 cell line (Supplementary Fig. S2d). To figure out the role of SIRT7 in cell proliferation in long-term upon stress, colony formation assay was employed, which also supported the results of CCK8 and flow cytometry assays in A2058 and A375 melanoma cell lines (Fig. 2c; Supplementary Fig. S2e). Besides, we also employed 451Lu melanoma cell line for the verification of the role of SIRT7 in melanoma, and it revealed that SIRT7 deficiency amplified cell death resulted from the treatment with TM or HBSS (Supplementary Fig. S3a–c). The expressions of cleaved PARP and cleaved caspase-3 were up-regulated more significantly after the knockdown of SIRT7 upon TM or HBSS treatment (Fig. 2d). In contrast, overexpression of SIRT7 in WM35 cell line reversed the up-regulation of cleaved-PARP and cleaved caspase-3 caused by TM (Fig. 2d). Besides, we also found that the deficient of SIRT7 expression led to prominent impairment of cell viability and increase of cell apoptosis under hypoxic condition (Supplementary Fig. S3d, e). Collectively, these data proved that SIRT7 acted as a cyto-protective factor that enabled melanoma cell survival under either ER stress or nutrient deprivation. It should be noted that SIRT7 was prominently up-regulated in metastatic melanoma compared with primary melanoma, which prompted us to see whether SIRT7 had an impact on melanoma cell invasion or migration. Transwell assay proved that SIRT7 deficiency showed little effect on the invasion or migration of melanoma cells (Supplementary Fig. S4a, b), indicating that SIRT7 probably did not play a role in regulating melanoma metastasis.

To evaluate the in vivo relevance of the above results, we employed a pre-clinical xenograft tumor model in nude mice via subcutaneous injection with A2058 melanoma cell with or without the stable knockdown of SIRT7 by lentivirus transfection (Supplementary Fig. S4c). The knockdown of SIRT7 induced prominent delay of tumor growth, with the tumor weight and tumor volume significantly down-regulated in comparison to the control (Fig. 2e). Through immunofluorescence staining analysis in tumors of the control group, we found that HSPA5 staining intensity was accentuated in the central region (poor blood supply) compared to the peripheral region (normal blood supply) (Fig. 2f; Supplementary Fig. S4d). Meanwhile, SIRT7 staining intensity revealed a similar trend as that of HSPA5, supporting it as a stress-responsive factor in vivo (Fig. 2f; Supplementary Fig. S4d). Furthermore, tumors with SIRT7 deficiency showed reduced Ki67 expression and up-regulated cleaved caspase-3 level (Fig. 2g; Supplementary Fig. S4f), heralded that SIRT7 enabled tumor growth by inhibiting cell apoptosis.

### SIRT7 selectively activates the IRE1α-XBP1 branch of UPR and downstream ERK pathway

**Fig. 3:**
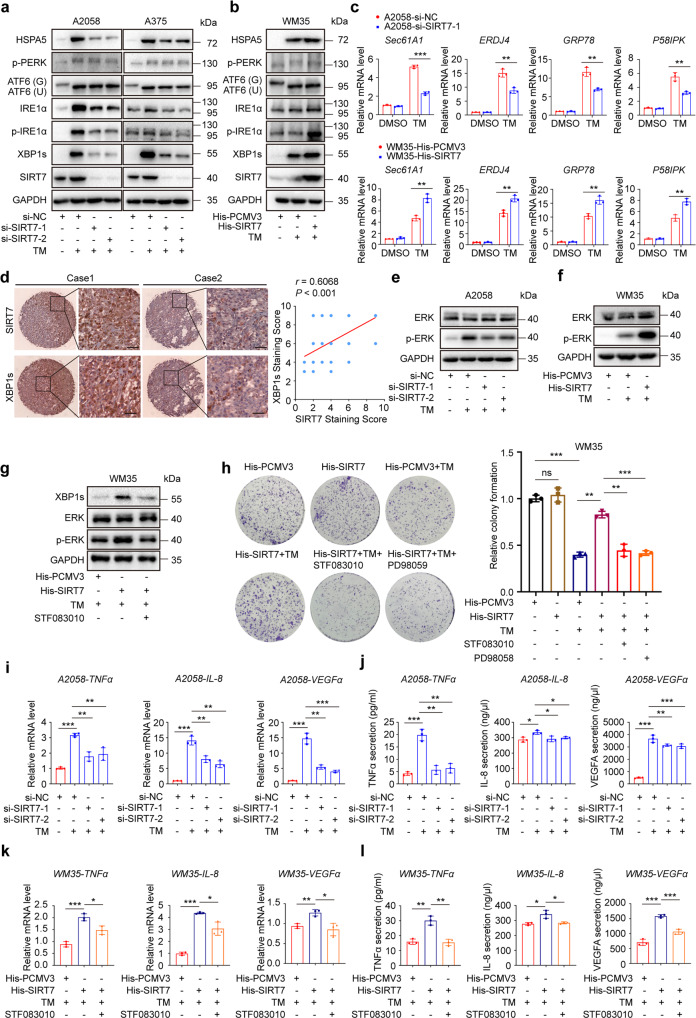
SIRT7 selectively activates IRE1α-XBP1 branch of UPR and downstream ERK pathway. **a**, **b** Immunoblotting analysis the expressions of three main branches of UPR in A2058, A375 and WM35 cells with indicated treatment. ATF6 (G), Glycosylated-ATF6; ATF6 (N), Non-glycosylated-ATF6. **c** Relative mRNA level of XBP1 downstream target genes in A2058 and WM35 cells with indicated treatment. **d** Immunohistochemical staining and correlation analysis of SIRT7 and XBP1s in TMA. Scale bar, 100 μm. **e**, **f** Immunoblotting analysis of ERK and phosphor-ERK in A2058 and WM35 cells with indicated treatment. **g** Immunoblotting analysis of XBP1s, ERK and phosphor-ERK in SIRT7-overexpressed WM35 treated with TM (3 μM) and STF083010 (50 μM) for 24 h. **h** Colony formation of SIRT7-overexpressed WM35 cells treated with TM (3 μM), STF083010 (50 μM) or PD98059 (10 μM) for 24 h. **i**, **j** Relative mRNA level and ELISA analysis of tumor-promoting cytokines in A2058 cells with indicated treatment. **k**, **l** Relative mRNA and ELISA analysis of tumor-promoting cytokines in WM35 cells with indicated treatment. Data represent the mean ± SD of triplicates. *r* value was calculated by Spearman correlation*. P* value was calculated by two-tailed Student’s *t*-test. *P < 0.05, **P < 0.01, ***P < 0.001. ns non-significant

The adaptive activation of UPR is a protective mechanism for cells undergoing ER stress and nutrient deprivation.^39^ We thus asked whether SIRT7 enabled melanoma cell survival via the regulation of UPR under stress. Upon the pharmacological ER stress inducer TM, three main branches of UPR were all prominently activated, as revealed by up-regulated expression of ATF6, increased phosphorylation of PERK, increased expression and phosphorylation of IRE1α, and potentiated spliced form of IRE1α downstream factor XBP1 (Fig. 3a). Nevertheless, we noticed that the knockdown of SIRT7 had little impact on ATF6 expression or PERK phosphorylation, but led to drastic down-regulation of IRE1α expression, IRE1α phosphorylation and spliced XBP1 expression, so was XBP1 downstream HSPA5 (Fig. 3a). In parallel, the overexpression of SIRT7 in WM35 melanoma cell line resulted in up-regulation of IRE1α expression, IRE1α phosphorylation, spliced XBP1 expression, and HSPA5 expression caused by TM treatment, but marginal influence on ATF6 and phosphorylated-PERK (Fig. 3b). We additionally employed qRT-PCR assay to examine the alterations of XBP1 transcriptional target genes SEC61 translocon subunit alpha 1 (*Sec61A1)*, DnaJ heat shock protein family (Hsp40) member B9 (*ERDJ4)*, glucose regulated protein 78 (*GRP78)*, and 58 kD inhibitor of PKR (*p58IPK)*. The knockdown of SIRT7 prominently impaired TM-induced up-regulation of these molecules in A2058 cell line, whereas SIRT7 overexpression could amplify the up-regulation of these targets in WM35 cell line (Fig. 3c). Apart from this, immunoblotting analysis revealed that the knockdown of SIRT7 had a marginal impact on neither the expressions of HSPA5, IRE1α, ATF6, XBP1s, phosphor-PERK nor phosphor-IRE1α under normal condition (Supplementary Fig. S5a), suggesting that SIRT7 is dispensable for the activation of UPR under normal condition. Thereafter, immunohistochemical staining analysis in TMA revealed that XBP1s staining intensity was prominently higher in melanoma than in nevus (Supplementary Fig. S5b). There was a robust positive correlation between SIRT7 staining scores and XBP1s staining scores in melanoma tissues (Fig. 3d). Therefore, SIRT7 could selectively activate the IRE1α-XBP1 branch of UPR under stress.

As a stress sensor, it has been reported that IRE1α could also induce the activation of MAPK pathways including ERK, JNK, and P38 MAPK under ER stress,^24,25^ among which ERK and JNK could play a pro-survival role. Immunoblotting analysis revealed that TM robustly induced the phosphorylation of ERK, JNK, and p38 MAPK in A2058 melanoma cells (Supplementary Fig. S5c). This effect could be blocked by the treatment with IRE1α specific inhibitor 4μ8C or STF083010 (Supplementary Fig. S5c), confirming that the activation of MAPK pathways under ER stress was largely attributed to IRE1α. Intriguingly, it was found that the knockdown of SIRT7 only impaired the activation of ERK after the treatment with TM, whereas displayed little effect on either the phosphorylation of JNK or p38 MAPK in A2058 melanoma cell (Fig. 3e; Supplementary Fig. S5d). In parallel, the overexpression of SIRT7 in WM35 melanoma cell also induced the activation of ERK, while a marginal impact on JNK or p38 MAPK activation was observed (Fig. 3f; Supplementary Fig. S5e). Further, we treated SIRT7-overexpressed WM35 melanoma cell with IRE1α-specific inhibitor STF083010 under ER stress and found that STF083010 could reverse the SIRT7 overexpression-mediated ERK activation and XBP1s up-regulation (Fig. 3g), which suggested that the activation of the ERK by SIRT7 was in an IRE1α-dependent manner. The selective regulation of ERK rather than JNK or p38 MAPK after the intervention of SIRT7 under stress might be due to different characteristics of IRE1α function between melanoma cells and other types of cells. To prove that the selective activation of the IRE1α-XBP1 axis and IRE1α-dependent ERK activation accounted for the protective role of SIRT7 under stress, we employed colony formation assay by using WM35 melanoma cells with or without SIRT7 overexpression. As was revealed, while SIRT7 overexpression significantly restored cell survival under the treatment of TM, co-treatment with either IRE1α inhibitor STF083010 or ERK inhibitor PD98059 prominently abolished the protective effect of SIRT7 (Fig. 3h).

IRE1α could also regulate the expression and secretion of cytokines via XBP1 to promote tumor cell survival under ER stress, especially tumor-promoting factors like TNFα, IL-8, and VEGFα.^40,41^ Therefore, we wondered whether SIRT7 also regulated these cytokines with oncogenic function. While TM treatment robustly elevated mRNA levels and secretion of TNFα, IL-8, and VEGFα, the knockdown of SIRT7 prominently negated this alteration (Fig. 3i, j). In parallel, overexpression of SIRT7 in WM35 melanoma cell promoted the mRNA levels and secretion of TNFα, IL-8, and VEGFα under ER stress (Fig. 3k, l). Of note, co-treatment with IRE1α inhibitor STF083010 dampened the facilitative effect of SIRT7 on either the expression or the secretion of TNFα, IL-8, and VEGFα under TM treatment (Fig. 3k, l). Therefore, SIRT7 could also initiate IRE1α-dependent secretion of TNFα, IL-8 and VEGFα to mediate oncogenesis under the stressful circumstances.

### SIRT7 de-acetylates SMAD4 to regulate IRE1α transcription

**Fig. 4:**
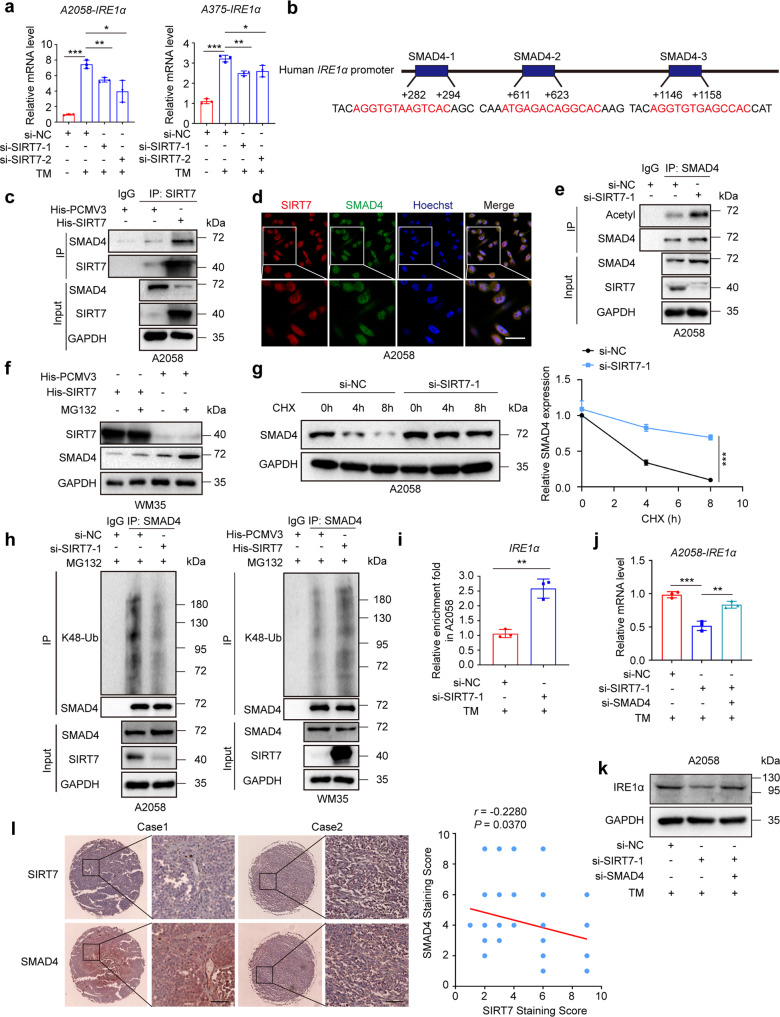
SIRT7 de-acetylates SMAD4 to regulate IRE1α transcription. **a** Relative mRNA level of *IRE1α* in A2058 and A375 cells with indicated treatment. **b** Three SMAD4 binding sites were identified in *IRE1α* promoter through transcription factor analysis. **c** Co-immunoprecipitation analysis of complex formation between endogenous SMAD4 and His-tagged SIRT7 immunoprecipitated from A2058 cells with indicated treatment. **d** Immunofluorescence staining of SIRT7 and SMAD4 in A2058 cells. Scale bar, 50 μm. **e** Co-immunoprecipitation analysis of acetyl-SMAD4 in A2058 cells with or without the knockdown of SIRT7. **f** Immunoblotting analysis of SMAD4 and SIRT7 in WM35 cells with or without SIRT7 overexpression treated with MG132 (10 μM) for 6 h. **g** Immunoblotting analysis of SMAD4 in A2058 cells with or without the knockdown of SIRT7 pre-treated with CHX (50 μg/mL) for 0 h, 4 h and 8 h. **h** Co-immunoprecipitation analysis of interaction between SMAD4 and K48-linked ubiquitin chain in A2058 cells with or without the knockdown of SIRT7 and WM35 cells with or without SIRT7 overexpression with MG132 treatment. **i** Chromatin immunoprecipitation analysis of the enrichment of SMAD4 to the promoter of *IRE1α* in A2058 cells with indicated treatment. **j** Relative mRNA level of *IRE1α* in A2058 cells with or without the knockdown of SIRT7 or SMAD4 under the treatment with TM (3 μM) for 24 h. **k** Immunoblotting analysis of IRE1α in A2058 cells with or without the knockdown of SIRT7 or SMAD4 treated with TM (3 μM) for 24 h. **l** Immunohistochemical staining and correlation analysis of SIRT7 and SMAD4 in TMA. Scale bar, 100 μm. Data represent the mean ± SD of triplicates. *r* value was calculated by Spearman correlation*. P* value was calculated by two-tailed Student’s *t*-test. *P < 0.05, **P < 0.01, ***P < 0.001

We previously proved that SIRT7 selectively activated the IRE1α-XBP1 axis to simultaneously regulate downstream ERK signal and secretion of cytokines, thus enabling melanoma cell survival under ER stress. Of note, the mRNA of IRE1α was prominently reduced after the knockdown of SIRT7 in TM-treated melanoma cells (Fig. 4a), indicating that the regulation of IRE1α by SIRT7 occurred at the transcriptional level. hTFtarget analysis revealed the potential binding sites of transcriptional factors to *IRE1α* promoter, and SMAD4 was the candidate upstream regulator (Fig. 4b). More intriguingly, SIRT7 has been documented to antagonize TGF-β signaling via direct deacetylation and degradation of SMAD4.^42^ In melanoma cell, co-immunoprecipitation (co-IP) assay confirmed the direct interaction between SIRT7 and SMAD4 (Fig. 4c). Immunofluorescence staining assay also proved the co-localization between SIRT7 and SMAD4 in nucleus (Fig. 4d). Subsequent co-IP assay revealed that the knockdown of SIRT7 led to increased interaction between actyl- group and SMAD4 (Fig. 4e), concurrently up-regulated protein level of SMAD4 (Fig. 4e). After the treatment with proteasome inhibitor MG132, the reduction of SMAD4 protein level caused by SIRT7 overexpression was prominently reversed (Fig. 4f). Cycloheximide (CHX) pulse-chase analysis manifested that the turnover of intracellular SMAD4 was impaired in cells with SIRT7 deficiency (Fig. 4g). Further co-IP analysis showed that the interaction between K48-linked ubiquitin chain and SMAD4 was impaired in A2058 melanoma cells with SIRT7 deficiency, whereas significantly increased in WM35 melanoma cells after the overexpression of SIRT7 (Fig. 4h). Moreover, we also examined the interaction between K63-linked ubiquitin chain and SMAD4 after the intervention of SIRT7 in either A2058 or WM35 melanoma cells, and there was no prominent alteration (Supplementary Fig. S5f). Therefore, SIRT7 directly de-acetylated SMAD4 and triggered its degradation in a ubiquitin-proteasome system-dependent way.

Under ER stress, SMAD4 expression was significantly up-regulated in melanoma cells with SIRT7 deficiency. Concurrent chromatin immunoprecipitation (ChIP) assay displayed more enrichment of SMAD4 to the promoter of *IRE1α* (Fig. 4i). We used siRNA against *SMAD4* to obtain the knockdown of its expression (Supplementary Fig. S5g), which reversed SIRT7 deficiency-induced down-regulation of IRE1α (Fig. 4j, k). Besides, the enrichment of SMAD4 to the promoter of *IRE1α* also displayed consistent alteration (Supplementary Fig. S5h). Moreover, immunohistochemical analysis in TMA revealed that the staining intensity of SMAD4 was down-regulated in melanoma compared to nevus (Supplementary Fig. S5i), and the staining intensities of SIRT7 were negatively correlated with that of SMAD4 (Fig. 4l). Collectively, our data proposed the model that SIRT7 deacetylated SMAD4 to trigger its ubiquitination and degradation, thereby relieved the transcriptional suppression on IRE1α to activate IRE1α-XBP1 axis and defend against ER stress in melanoma.

### SIRT7 up-regulation compromises anti-tumor immunity by promoting PD-L1 via the IRE1α-XBP1 axis

**Fig. 5:**
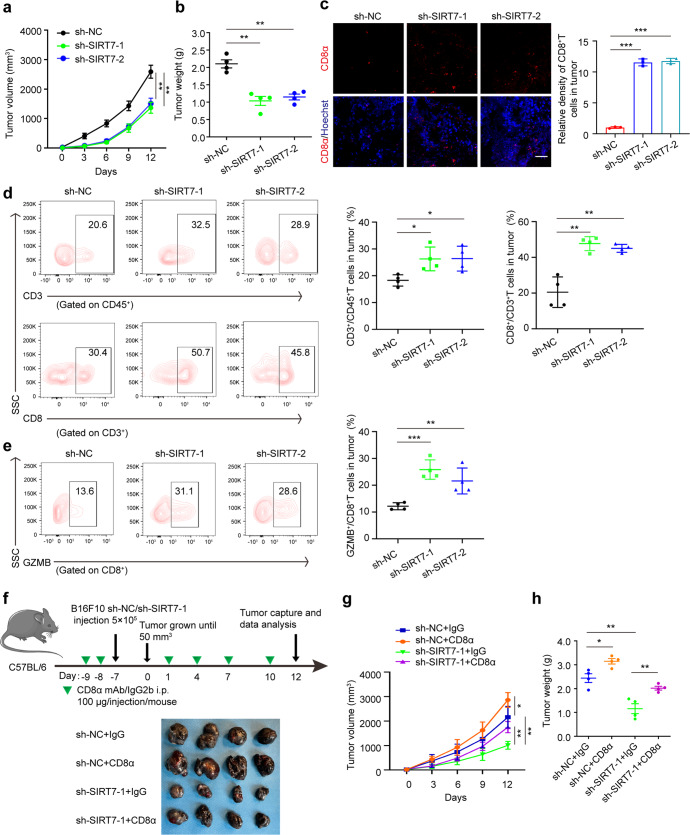
SIRT7 up-regulation eradicates anti-tumor immunity. **a**, **b** Tumor volumes and weights of B16F10 xenografts with or without the knockdown of SIRT7. **c** Immunofluorescence staining of CD8α in B16F10 xenografts with or without the knockdown of SIRT7. Scale bar, 50 μm. **d** FACS of CD3^+^in CD45^+^cells and CD8^+^ in CD3^+^ TILs from B16F10 xenografts with indicated treatment and corresponding quantification. **e** GZMB^+^CD8^+^ TILs from B16F10 xenografts with indicated treatment and corresponding quantification. **f** A schematic view of the treatment plan that C57BL/6 mice burdened with negative control (sh-NC) and SIRT7 shRNA Lentivirus (sh-SIRT7-1)-transfected B16F10 tumors with or without CD8α mAb treatment and images of isolated tumors from mice that with indicated treatment. Tumor volumes and weights in each group were calculated and displayed in (**g**) and (**h**). Symbols of one dot indicates one mouse, and the error bars are mean with ±S.D. *P* value was calculated by two-tailed Student’s *t*-test. *P < 0.05, **P < 0.01, ***P < 0.001

In addition to the previous pre-clinical xenograft tumor model, we also subcutaneously injected B16F10 melanoma cells with or without SIRT7 deficiency into immune-competent C57B6/L mice to see the effect on tumor growth (Supplementary Fig. S6a, b). As a result, the knockdown of SIRT7 similarly induced prominent delay of tumor growth in C57B6/L mice (Fig. 5a, b; Supplementary Fig. S6c). Unexpectedly, both the immunofluorescence staining and flow cytometry analysis unveiled more infiltration of CD8^+^T cells in SIRT7-deficient tumors compared to control, and so was the infiltration of GzmB-positive CD8^+^T cells (Fig. 5c–e). Moreover, we also employed immunohistochemical staining analysis in TMA to examine the relationship between tumorous SIRT7 expression and CD8^+^T cells infiltration, which revealed the negative correlation between SIRT7 and CD8 in melanoma tissues (Supplementary Fig. S6d). These results indicated that SIRT7 knockdown might suppress melanoma progression by activating CD8^+^T cell-dependent anti-tumor immunity. To prove this, we employed systemic administration of CD8α antibody to eliminate CD8^+^T cells (Supplementary Fig. S6e). As a result, the suppressive role of SIRT7 knockdown on melanoma growth was partially abrogated as shown by the alterations of tumor volumes and tumor weights (Fig. 5f–h), proving that SIRT7 up-regulation contributed to melanoma growth by disrupting CD8^+^T cells-mediated immunosurveillance. Apart from CD8^+^T cells, other types of immune cells like macrophages, dendritic cells (DCs), myeloid-derived suppressor cells (MDSCs), and regulatory T cells (Tregs) are also greatly implicated in the regulation of anti-tumor immunity.^43–45^ Through flow cytometry analysis of immune cells in B16F10 implanted tumor, we found that the infiltration of macrophage was prominently increased after the knockdown of SIRT7 in B16F10 xenograft tumor (Supplementary Fig. S6f), whereas the quantity of either dendritic cells or MDSCs (including both M-MDSCs and PMN-MDSCs) was not significantly altered after the knockdown of SIRT7 (Supplementary Fig. S6g, h). Moreover, the infiltration of Tregs was merely detectable in the B16F10 xenograft tumor according to our flow cytometry analysis (data not shown). Therefore, in addition to CD8^+^T cells, the infiltration of macrophages might be also implicated in mediating SIRT7-dependent immune evasion.

We also examined the role of SIRT7 in anti-tumor immunity under stress in vivo. B16F10 melanoma cells were subcutaneously implanted into C57BL/6 mice with or without the stable knockdown of SIRT7, and the mice then received the intraperitoneal injection with HA15 to trigger ER stress that mimics the stressful TME (Supplementary Fig. S7a).^46^ Flow cytometry analysis was employed to analyze the immune cells in implanted tumors, including CD8^+^T cells, macrophages, MDSC DCs, and Tregs. As a result, in response to systemic HA15 administration, the deficiency of tumorous SIRT7 prominently delayed the growth of implanted B16F10 tumor (Supplementary Fig. S7a–c). Moreover, the infiltration of CD8^+^T cells was significantly increased (Supplementary Fig. S7d), in parallel with the increase in the percentage of GzmB-positive CD8^+^T cells (Supplementary Fig. S7e). Apart from CD8^+^T cells, the infiltration of macrophage was prominently increased (Supplementary Fig. S7f), whereas the quantities of dendritic cells (Supplementary Fig. S7g) and MDSCs (including both M-MDSCs and PMN-MDSCs) were not significantly altered (Supplementary Fig. S7h). Therefore, CD8^+^T cells and macrophages were also greatly implicated in tumorous SIRT7-mediated anti-tumor immunity under ER stress in melanoma.

**Fig. 6:**
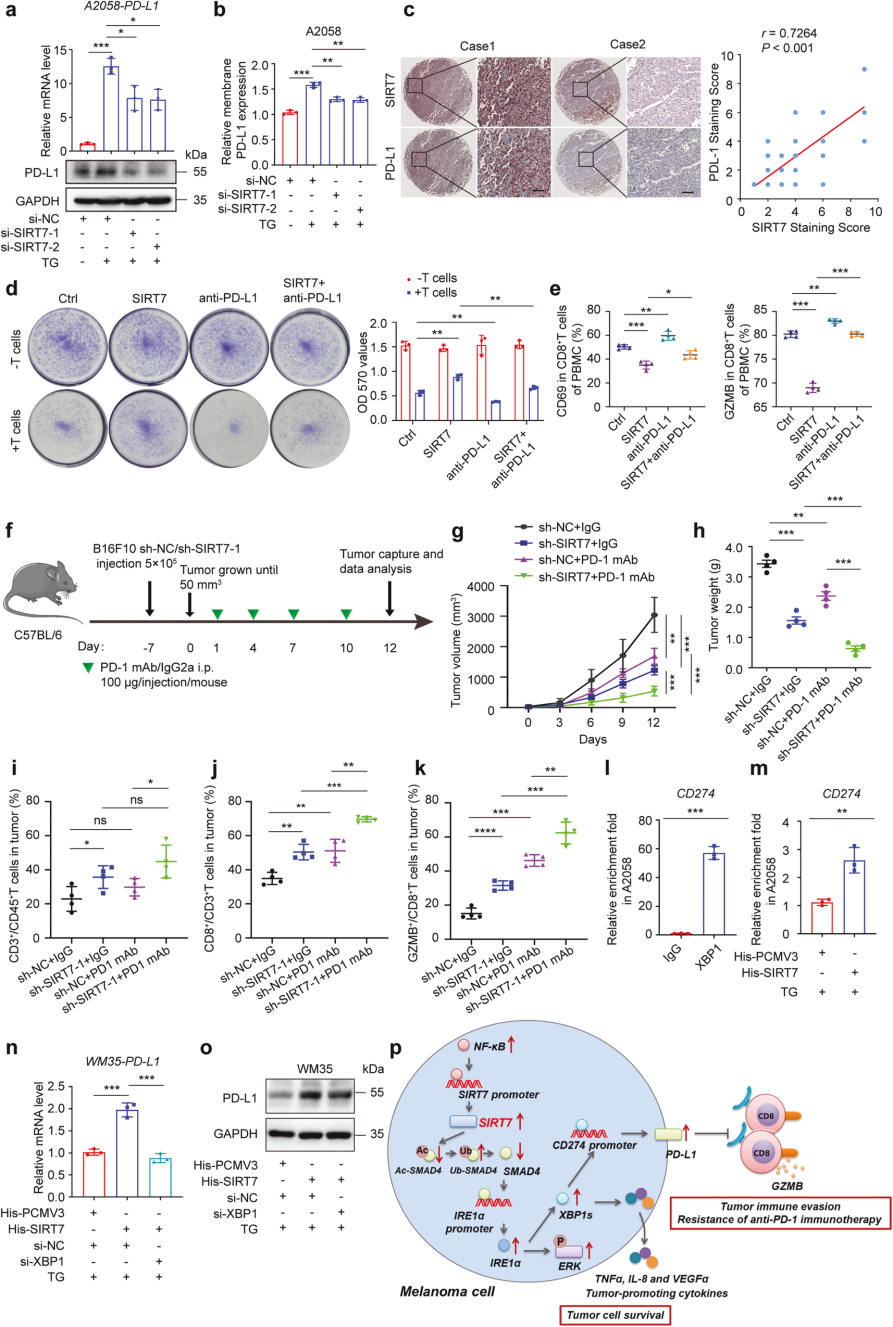
SIRT7 promotes PD-L1 expression via IRE1α-XBP1 axis. **a** Relative mRNA and protein levels of PD-L1 in A2058 cells with indicated treatment. **b** Relative membrane expression of PD-L1 in A2058 cells with indicated treatment. **c** Immunohistochemical staining and correlation analysis of SIRT7 and PD-L1 in TMA. Scale bar, 100 μm. *r* value was calculated by Spearman correlation. **d** Co-culture of A2058 cells transfected with control or SIRT7 overexpression plasmid with TG treatment and activated T cells (1:3) for 24 h with or without the treatment with PD-L1 neutralizing antibody (Avelumab, 20 μg/mL) for 8 h. Cytotoxicity was then quantified by a spectrometer at OD (570 nm) followed by crystal violet staining. **e** FACS of CD69^+^in CD8^+^T cells and GZMB^+^CD8^+^ TILs from co-culture system with indicated treatment and corresponding quantification. **f** A schematic view of the treatment plan that C57BL/6 mice burdened with negative control (sh-NC) and sh-SIRT7-1-transfected B16F10 tumors with or without PD-1 mAb treatment as indicated. Tumor volumes and weights in each group were calculated and displayed in (**g**) and (**h**). The quantification of FACS of CD3^+^in CD45^+^cells (**i**), CD8^+^ in CD3^+^ TILs (**j**) and GZMB^+^CD8^+^ TILs (**k**) from B16F10 xenografts with indicated treatment. Symbols of one dot indicates one mouse, and the error bars are mean with ±S.D. **l** Chromatin immunoprecipitation analysis of the enrichment of XBP1 to the promoter of *CD274* in A2058 cells. **m** Chromatin immunoprecipitation analysis of the enrichment of XBP1 to the promoter of *CD274* in WM35 cells with or without SIRT7 overexpression under the treatment with TG (1 μM) for 24 h. **n**, **o** Relative mRNA and protein levels of PD-L1 in WM35 cells with indicated treatment. Data represent the mean ± SD of triplicates. **p** A schematic model summarizing how SIRT7 orchestrates melanoma progression by simultaneously promoting tumor cell survival and immune evasion via the activation of unfolded protein response. Under ER stress condition, NF-κB p65 contributes to SIRT7 up-regulation in melanoma. SIRT7 directly interacts with and de-acetylates SMAD4 to promote the transcription of IRE1α. Then, SIRT7 promotes melanoma cell survival by selective activation of IRE1α-XBP1 branch of UPR and downstream ERK pathway. Apart from the direct effect on tumor cell survival, SIRT7 up-regulation also eradicates anti-tumor immunity by promoting PD-L1 expression via IRE1α-XBP1 axis. Based on this, the knockdown of SIRT7 prominently increases the treatment efficacy of anti-PD-1 antibody by potentiating CD8^+^T cells-dependent anti-tumor immunity. Data represent the mean ± SD of triplicates. *P* value was calculated by two tailed Student’s *t*-test. *P < 0.05, **P < 0.01, ***P < 0.001. ns non-significant

Recent cumulative evidence has pointed out that multiple immune checkpoints expressed by tumor cells play crucial roles in tumor immune evasion, among which PD-L1/PD-1 is regarded as a pivotal pair of valuable therapeutic targets. The blockade of PD-L1/PD-1 is in position to disrupt the interaction between tumor cells and tumor-specific T cells, which can help to restore tumor-specific immune response.^47^ Surprisingly, while the knockdown of SIRT7 displayed a marginal effect on both the mRNA and protein levels of PD-L1 under normal conditions (Supplementary Fig. S8a), the deficiency of SIRT7 expression restrained TG-induced up-regulation of PD-L1 at both transcriptional and protein levels (Fig. 6a). Moreover, membrane PD-L1 expression was prominently reduced in SIRT7-deficient melanoma cells compared with control under ER stress (Fig. 6b; Supplementary Fig. S8b). The immunohistochemical staining analysis in TMA also revealed that the intensity of SIRT7 was in significantly positive correlation with that of PD-L1, further supporting the regulatory relationship between SIRT7 and PD-L1 in melanoma (Fig. 6c). It should be noted that IFN-γ derived from infiltrated CD8^+^T cells is a cardinal trigger of PD-L1 expression in tumor cell,^48^ which prompted us to examine whether SIRT7 affects IFN-γ-driven PD-L1 up-regulation. Through qRT-PCR and immunoblotting analysis, while IFN-γ treatment robustly induced the increase of both mRNA and protein levels of PD-L1, the overexpression of SIRT7 and concurrent knockdown of XBP1 displayed little impact, suggesting that SIRT7 does not affect IFN-γ-driven PD-L1 up-regulation in melanoma (Supplementary Fig. S8c).To confirm whether tumorous SIRT7 modulated the cytotoxic function of CD8^+^T cells via the regulation of PD-L1 expression, T cell-mediated tumor cell-killing assay was employed as previously described.^49^ As was revealed, the overexpression of SIRT7 conferred the resistance of melanoma cells pre-treated with TG to T cell-mediated killing effect (Fig. 6d). In addition, PD-L1 antibody co-treatment prominently abrogated the protective effect of SIRT7 overexpression on tumor cells pre-treated with TG in response to T cell-mediated killing function (Fig. 6d). After the overexpression of SIRT7, the expression of CD69 and Granzyme B in CD8^+^T cells were both prominently mitigated, and this alteration trend could be reversed after the treatment with anti-PD-L1 antibody in co-culture system (Fig. 6e; Supplementary Fig. S8d). Therefore, tumorous SIRT7 eradicated CD8^+^T cell-dependent immune surveillance in a PD-L1-dependent way.

The up-regulation of PD-L1 is an adaptive mechanism mediating the resistance to anti-PD-1 immunotherapy.^50^ Since the knockdown of SIRT7 can lead to more CD8^+^T cells infiltration in TME and induce prominent down-regulation of PD-L1, we proposed that SIRT7 deficiency could synergize with anti-PD-1 immunotherapy in melanoma. To this end, we established the pre-clinical tumor model via subcutaneously implanting B16F10 melanoma cells into C57BL/6 mice with or without the knockdown of SIRT7 and the mice then received the intraperitoneal injection with anti-PD-1 antibody (Fig. 6f). The knockdown of SIRT7 could prominently increase the efficacy of anti-PD-1 antibody (Fig. 6g, h; Supplementary Fig. S8e). In addition, the combination of the knockdown of SIRT7 and anti-PD-1 antibody treatment could induce more infiltration of CD8^+^T cells and GzmB-positive CD8^+^T cells in the implanted tumor (Fig. 6i–k; Supplementary Fig. S8f), indicating that the combination treatment approach triggered more prominent activation of anti-tumor immunity. Of note, the combination of systemic anti-PD-1 antibody treatment and the suppression of tumorous SIRT7 expression displayed little impact on the weight of mice (Supplementary Fig. S8g), suggesting that this combinatorial therapeutic approach would not significantly impact the overall metabolic status of mice.

We then wondered about the mechanism underlying the regulation of PD-L1 expression by SIRT7. hTFtarget analysis listed XBP1 as a candidate upstream regulator of *CD274* (encoding PD-L1) (Supplementary Fig. S9a), which was then proved by ChIP assay (Fig. 6l). ChIP assay showed that overexpression of SIRT7 facilitated the enrichment of XBP1 to the promoter region of *CD274* mRNA under TG treatment induced-ER stress (Fig. 6m). We treated SIRT7-overexpressed melanoma cell with IRE1α inhibitor STF083010 or the transfection with siRNA against *XBP1*, which displayed that the up-regulation of PD-L1 by SIRT7 was significantly suppressed at both mRNA and protein levels under ER stress (Fig. 6n, o; Supplementary Fig. S9b). Furthermore, concerning SIRT7-regulated PD-L1 expression via the IRE1α-XBP1 axis, we pre-treated SIRT7-overpressed melanoma cells with IRE1α inhibitor STF083010 and XBP1 inhibitor Toyocamycin respectively in the co-culture system to see whether IRE1α and XBP1 participate in endowing SIRT7-overpressed melanoma cells the capacity to mitigate the function of CD8^+^T cells. As was revealed by flow cytometry analysis, the percentage of CD69 and Granzyme B that can reflect the functional status of CD8^+^T cells were both prominently mitigated after the overexpression of SIRT7, whereas the alteration trend could be significantly reversed by pharmacological inhibition of either IRE1α or XBP1 (Supplementary Fig. S9c). Taken together, SIRT7 activated the IRE1α-XBP1 axis to facilitate PD-L1 transcription and thereby eradicate CD8^+^T cell-mediated immunosurveillance.

Of note, it has been previously demonstrated that the knockdown of SIRT7 can promote the acetylation of myocyte enhancer factor 2D (MEF2D), and increase its binding, along with acetylated histones, to the promoter region of *CD274*, promoting the transcription and expression of PD-L1.^20^ We wondered whether the regulation of PD-L1 by SIRT7 was also associated with MEF2D under ER stress. As was revealed by the co-immunoprecipitation experiment, there was no significant change of MEF2D acetylation after the knockdown of SIRT7 under ER stress (Supplementary Fig. S9d). In addition, the knockdown of MEF2D displayed a marginal effect on PD-L1 expression in SIRT7-overexpressed melanoma cells under TG treatment (Supplementary Fig. S9e). Therefore, the regulation of PD-L1 by SIRT7 is independent of MEF2D, and the regulatory mechanism of MEF2D by SIRT7 does not exist in melanoma cells under ER stress.

### NF-κB p65 contributes to SIRT7 up-regulation in melanoma

Ultimately, we investigated the mechanism underlying SIRT7 up-regulation in melanoma. Since our genomic analysis in the TCGA SKCM database and qRT-PCR assay in melanoma cell lines both pointed out that the up-regulation of SIRT7 occurred at the transcriptional level, we thus went on to figure out the upstream transcriptional factor responsible for this. hTFtarget revealed that NF-κB p65 was among those candidate upstream regulators binding the promoter of *SIRT7* (Fig. S10a). ChIP assay also confirmed the enrichment of NF-κB p65 to the promoter region of *SIRT7* mRNA (Fig. S10b). Pharmacological inhibition of NF-κB p65 by pyrrolidinedithiocarbamate ammonium (APDC) led to reduced phosphorylation of p65, paralleled with significant down-regulation of SIRT7 mRNA and protein levels (Fig. S10c, d), indicating that NF-κB p65 was responsible for SIRT7 up-regulation in melanoma under normal condition. In response to TM treatment, the phosphorylation of NF-κB p65 was robustly induced (Fig. S10c, d). In accordance, the enrichment of NF-κB p65 to the promoter of *SIRT7* was enhanced (Fig. S10e). We treated TM-stimulated melanoma cells with NF-κB p65 inhibitor APDC, and the up-regulation of SIRT7 was prominently impaired (Fig. S10c, d), raising the notion that NF-κB p65 also contributed to SIRT7 up-regulation under stress. Further co-expression analysis in the TCGA SKCM database unveiled the positive correlation between SIRT7 mRNA and p65 mRNA levels (Fig. S10f). In line with this, immunohistochemical staining analysis also displayed a positive correlation between SIRT7 expression and p-p65 expression (Fig. S10g), providing in vivo evidence of the regulatory relationship between NF-κB p65 and SIRT7 in melanoma. Therefore, NF-κB p65 contributes to SIRT7 up-regulation under stress in melanoma via transcriptional regulation.

## Discussion

Herein, we first identified that SIRT7 was a stress-responsive factor and its expression was significantly increased in melanoma. Then, we proved that SIRT7 enabled melanoma cell survival under stress, and the selective activation of the IRE1α-XBP1 branch of UPR and downstream ERK pathway driven by SIRT7 up-regulation was responsible for this effect. Mechanistically, SIRT7 directly interacted with and de-acetylated SMAD4 to promote its ubiquitination and degradation, which alleviated the transcriptional repression on IRE1α. Apart from the direct effect on tumor cell survival, SIRT7 up-regulation also repressed anti-tumor immunity by promoting PD-L1 expression via the IRE1α-XBP1 axis. Based on this, the knockdown of SIRT7 could prominently increase the treatment efficacy of anti-PD-1 antibody by potentiating CD8^+^T cell-dependent anti-tumor immunity. Ultimately, it was proved that NF-κB p65 contributed to SIRT7 up-regulation in melanoma under stress (Fig. 6p). Taken together, our data demonstrated that SIRT7 orchestrated melanoma progression by simultaneously promoting tumor cell survival and immune evasion via the selective activation of the IRE1α-XBP1 axis. Targeting SIRT7 could be employed as a promising strategy to restrain tumor growth and increase immunotherapy efficacy in melanoma.

SIRT7 is a versatile nuclear-localized NAD^+^-dependent deacetylase with multiple biological functions, and the dysregulation of SIRT7 expression is greatly implicated in the pathogenesis of different types of cancers. Initial investigation in human hepatocellular carcinoma revealed high expression of SIRT7 and its oncogenic potential in cancer.^51^ The de-acetylation of USP39 by SIRT7 can promote its stability and thereby accelerate HCC cell proliferation and tumorigenesis.^52^ Thereafter, it was proved that the up-regulation of SIRT7 expression exhibits oncogenic properties in colorectal cancer and serves as a valuable biomarker for predicting patients’ prognosis.^53^ In addition, SIRT7 also plays oncogenic roles in thyroid cancer and prostate cancer via DBC1/SIRT1 axis-mediated activation of Akt and p70S6K1 and the androgen receptor-mediated activation of autophagy, respectively.^14,17^ In contrary to these reports, some studies have demonstrated that SIRT7 exerts tumor suppressive role. In particular, SIRT7 expression is significantly down-regulated in breast cancer lung metastases and can inhibit breast cancer metastasis by antagonizing TGF-β signaling.^42^ In colon cancer, SIRT7 directly deacetylases WDR77 to interfere with the WDR77-PRMT5 interaction and suppresses tumor cell proliferation.^19^ What’s more, SIRT7 plays an inhibitory role in oral squamous cell carcinoma metastasis by promoting SMAD4 de-acetylation and restraining the process of epithelial-to-mesenchymal transition.^18^ Therefore, the pathogenic role of SIRT7 in cancer is tumor-type specific in a context-dependent manner. Our present study proved that SIRT7 plays an oncogenic factor in melanoma by synergistically modulating tumor cell survival and anti-tumor immune surveillance, underpinning SIRT7 as a promising therapeutic target, as well as extending the spectrum of cancers that SIRT7 plays a pathogenic role in. Of note, while SIRT7 exerts a marginal impact on tumor cell proliferation, invasion, and migration in melanoma under normal conditions, it displays a prominent protective role in maintaining tumor cell survival under stressful circumstances. This discrepancy might result from the compensated up-regulation and activation of SIRT7 triggered by the increase of intracellular NAD^+^ levels under stress. The present results also indicate that the function of one specific factor that seems to be redundant under normal conditions can be revealed in response to exogenous stimuli during cancer progression. Besides, it has been reported that SIRT7 is necessary for a global hypoacetylation of H3K18Ac that is associated with cellular transformation driven by the viral oncoprotein E1A, demonstrating that SIRT7-mediated H3K18 de-acetylation is critical in mediating cellular transformation programs.^10^ In this regard, the up-regulation of SIRT7 in melanoma compared with nevus indicates that SIRT7 might also drive the malignant transformation of melanocytes to become melanoma cells, in addition to its role in melanoma cell malignant characteristics. Of note, our genomic analysis in the TCGA SKCM database revealed that the increased transcription of SIRT7 might partially result from its ploidy status (gain and amplification), which is related to the expression status of SIRT7 under normal conditions. On the other hand, we proved that SIRT7 expression could be induced by multiple paradigms of exogenous stress, and the NF-κB p65 component contributed to not only the increase of SIRT7 transcription under normal conditions, but also the up-regulation of SIRT7 in response to stress. Therefore, both the copy number gain and NF-κB p65 are responsible for SIRT7 up-regulation under normal conditions, whereas NF-κB p65 plays a more critical role in SIRT7 up-regulation under stressful circumstances.

The proper activation of UPR is a critical protective mechanism upon ER stress and other exogenous stimuli. Melanoma cells suffer chronic ER stress due to the existence of oncogenic *BRAF*,^28^ and multiple mechanisms endow melanoma cells to be relatively resistant to ER stress and thereby have the growth advantage.^5,6,54^ While the molecular events downstream of UPR that enable melanoma cell survival under ER stress have been revealed in detail, how the activation of UPR is regulated in melanoma remains far from understood. A stressful microenvironment can induce the increase of NAD^+^ level, so we emphasized on NAD^+^-dependent sirtuins that might bring a linkage between exogenous stress and the activation of UPR. Compared to other family members of sirtuins, SIRT7 displays the most prominent up-regulation upon ER stress, and more importantly, it facilitates the activation of the IRE1α-XBP1 axis by attenuating the transcriptional suppression of IRE1α by SMAD4. Of note, the regulatory role of SIRT7 in UPR activation is highly selective for the IRE1α-XBP1 branch rather than PERK and ATF6 branches, whereas the underlying mechanism needs further investigation. In line with two previous reports,^18,42^ our data also proved that SIRT7 could directly interact with SMAD4 and induce its deacetylation. This conserved regulatory mechanism indicates that the regulation of UPR by SIRT7 via SMAD4 might also be similar in other types of cells and cancers. Apart from the protective effect on tumor cell survival, tumorous SIRT7 could also suppress CD8^+^T cell-dependent anti-tumor immunity to facilitate immune evasion. These two distinct biological effects share a similar mechanism that involves the activation of IRE1α. A previous study reported that SIRT7 formed a complex with MEF2D that suppressed PD-L1 expression in hepatocellular carcinoma,^20^ which was different from our present study that SIRT7 facilitated PD-L1 expression under ER stress in melanoma. This discrepancy might be due to not only the heterogeneity of different types of cancers, but also the underlying mechanism that directs the regulatory effect. While in hepatocellular carcinoma the regulation of PD-L1 by SIRT7 is dependent on MEF2D-mediated histone acetylation at the promoter region of *CD274*, the up-regulation of PD-L1 induced by SIRT7 is related to the activation of IRE1α-XBP1 axis and XBP1-driven transcriptional activation in melanoma, which is in consistent with the previous finding in macrophages.^27,55^ Our data has provided evidence that XBP1s can directly bind to the promoter region of *CD274* and induce its transcription, highlighting a novel linkage between stress signaling and anti-tumor immunity in cancer.

The application of approved anti-PD-1 antibodies in melanoma treatment has gained revolutionary progress in prolonging the patients’ prognosis.^56^ Nevertheless, low response rate and treatment resistance pose an obstacle in the path of improving the durable effect.^57,58^ Tumorous PD-L1 expression is tightly related to immunotherapy efficacy in melanoma,^59^ but the exact role remains controversial. For one thing, high expression of tumorous PD-L1 before anti-PD-1 antibody treatment is considered a valuable biomarker of a better response.^60^ For the other, sustained high expression of PD-L1 triggered by IFN-γ that was derived from re-activated CD8^+^ T cells upon anti-PD-1 antibody treatment would otherwise limit the treatment efficacy.^50,61^ In this regard, the role of tumorous PD-L1 in immunotherapy efficacy is highly related to the time phase of treatment (before or during the treatment), and to suppress the sustained PD-L1 up-regulation during treatment could help to elevate the therapeutic efficacy. Our present study has demonstrated that the up-regulation of SIRT7 is required for sustained PD-L1 expression in melanoma cells under the stressful circumstances, and targeting SIRT7 is a valuable strategy not only due to the direct suppressive effect on tumor growth via impairing tumor cell survival, but also the activation of CD8^+^T cells and anti-tumor immunity. In addition, the invigoration of tumor-infiltrating CD8^+^T cells by targeting tumorous SIRT7 and sustained PD-L1 expression can provide the cellular and molecular basis to potentiate the efficacy of anti-PD-1 immunotherapy. Of note, a novel inhibitor of SIRT7 (ID: 97491) is identified recently and it could significantly suppress the deacetylase activity of SIRT7 to impair tumor progression by increasing p53 stability.^62^ Moreover, two compounds named 2800Z and 40569Z respectively are proven to not only bind SIRT7 with high affinities instead of to other SIRT proteins but to induce apoptosis and increase the chemosensitivity to sorafenib in human liver cancer.^63^ Therefore, further pre-clinical studies and clinical trials involving SIRT7 inhibitors should be conducted to testify the translational potential of pharmacological inhibition of SIRT7 in treating melanoma, as well as the effect on immunotherapy efficacy.

The role of tumorous NF-κB in anti-tumor immunity is related with PD-L1 expression. NF-κB could regulate PD-L1 expression either directly at the transcriptional level or via alternative indirect mechanisms. Lim et al. has reported that NF-κB p65 could induce the expression of COP9 signalosome 5 (CSN5) that is required for TNF-α-mediated PD- L1 stabilization in cancer cells. Targeting CSN5 by curcumin can diminish p65-induced PD-L1 up-regulation and sensitize cancer cells to anti-CTLA-4 antibody.^64^ Besides, different binding sequences for NF-κB have been described on the promoter of the *PD-L1* gene,^65^ and NF-κB can directly promote PD-L1 expression at the transcriptional level to mediate immune evasion of cancer.^66^ Herein, we proved that stress-induced SIRT7 up-regulation was attributed to NF-κB p65-mediated transcriptional activation, and SIRT7 was required for tumorous PD-L1 expression up-regulation via the IRE1α-XBP1 axis under stress. Therefore, it can be proposed that NF-κB p65 might also indirectly promote PD-L1 expression via SIRT7 and its-mediated IRE1α-XBP1 axis, and this speculation might provide more evidence supporting the role of NF-κB p65 in anti-tumor immunity, which needs more investigation in the future.

In summary, our findings demonstrate that SIRT7 is a stress-responsive protective factor and plays an oncogenic role by simultaneously enabling tumor cell survival and immune evasion via the activation of unfolded protein response. Targeting SIRT7 might potentiate the anti-tumor capacity of cytotoxic CD8^+^T cells and increase the efficacy of anti-PD-1 immunotherapy. Given that SIRT7 is highly related to stress response, cytoprotective signaling, and immunology, our study provides the proof of a principle that the intervention of SIRT7 might bring progress in diseases associated with injury and inflammation, in addition to cancer.

## Materials and methods

### Materials

Tunicamycin (TM, ab120296) was purchased from Abcam (Cambridge, MA, USA). Thapsigargin (TG, T9033), Hydrogen Peroxide (H_2_O_2_, 88597), Hanks’ balanced salt solution (HBSS, H8264), and 2-deoxy-D-glucose (2-DG, D8375) were purchased from Merck Millipore (Billerica, MA, USA). 4μ8C (HY-19707), STF083010 (HY-15845), MG132 (S2619) was purchased from Selleck (Houston, USA), Cycloheximide (CHX, HY-12320), PD98059 (HY-12028), Pyrrolidinedithiocarbamate ammonium (APDC, HY-18738), human PD-L1 neutralizing antibody (Avelumab, HY-108730) and HA15 (HY-100437) were purchased from MedChemExpress (Monmouth Junction, NJ, USA).

### Cell culture and stable transfection

Human melanoma cell lines A375, A2058, SK-MEL-1 and WM35 were purchased from ATCC. 451Lu, UACC62 and UACC257 cells were kindly provided by Professor David Schrama (University Hospital Würzburg, Germany). FLFMM-34 was a metastatic melanoma cell line established in our lab.^67^ WM35, 451Lu and UACC257 cells were cultured in RPMI1640 with 10% FBS. FLFMM34, UACC62, A2058 and A375 cells were cultured in DMEM with 10% FBS. Human primary melanocytes (HPMs) were cultured in Medium 254 (Invitrogen) with 5% FBS and supplemented with Human Melanocyte Growth Supplement (Gibco). B16F10 melanoma cells (TCM2, Chinese Academy of Sciences, Shanghai, China) and PBMCs derived from healthy human donors were grown in RPMI 1640.

For stable knockdown, human SIRT7 shRNA Lentivirus (sh-hSIRT7-1 and sh-hSIRT7-2) and mouse SIRT7 shRNA Lentivirus (sh-mSIRT7-1 and sh-mSIRT7-2) were purchased from Shanghai GenePhama (China). The targeting sequences were described as follows: sh-hSIRT7-1: CCCUGAAGCUACAUGGGA AdTdT, sh-hSIRT7-2: CCUUUCUGU GAGAACGGAATT; sh-mSIRT7-1: GCGAGGAUCUGGUGACCGATT, sh-mSIRT7-2: CAGCUUCUAUCCCAGAUUATT. After 72 h of virus transduction, stable knockout A2058 cells and B16F10 cells were selected in DMEM and RPMI1640 medium respectively supplemented with puromycin (10 µg/mL) (Beyotime, China), and knockout efficiency was confirmed by immunoblotting.

### Flow cytometry analysis

After indicated treatment, melanoma cells were collected and detected the apoptosis ratio by staining with AnnexinV-FITC and PI following the instructions of Apoptosis Detection Kit I (Beyotime, Shanghai, China). For detection of PD-L1 expression on the cell membrane surface, melanoma cells were collected after indicated treatment and stained with anti-human CD274 (PD-L1) antibody (cat.329706, Biolegend). For detection of the efficiency of CD8^+^T cells depletion in mice, the peripheral blood samples were stained with PE anti-mouse CD4 and PE/Cyanine7 anti-mouse CD8α after removing red blood cells. For analysis of tumor-infiltrating immune cells, after the mice was sacrificed, tumors were obtained and grinded mechanically using a syringe plunger and filtered with a 70 μM filter filtration (FALCON cell strainer, Corning, NY, USA), and then the isolated single-cell suspensions were cultured in T cell medium (RPMI 1640) with cell activation cocktail (cat. 423304, Biolegend) for 5 h at 37 °C. Zombie UV (cat. 423108, Biolegend) was used for live/dead cell determination. Samples were blocked with anti-mouse Fc blocking Ab for 15 mins. Cells were washed and then surface stained with the following antibodies: Pacific Blue anti-mouse CD45, APC anti-mouse CD3, PE anti-mouse CD4, PerCP anti-mouse CD8α, PE anti-mouse/human CD11b, APC anti-mouse Ly-6C, PE/Cyanine7 anti-mouse Ly-6G, Brilliant Violet 650™ anti-mouse CD11c and APC anti-mouse F4/80. For Granzyme B staining, cells were fixed and permeabilized using True-Nuclear™ Transcription Factor Buffer Set (cat. 424401, Biolegend), and then stained with PE-Granzyme B anti-human/ mouse antibody. All staining cells were analyzed on BD LSRFortessa™ Flow Cytometer (BD Biosciences, San Jose, CA, USA). Data were analyzed using FlowJo v10 software (Treestar Inc) and GraphPad Prism 8.0 (GraphPad Software).

### Co-immunoprecipitation (co-IP) assay

Briefly, protein lysates were prepared in ice-cold RIPA buffer with phenylmethanesulfonyl fluoride (PMSF, Beyotime), and then incubated at 4 °C for 10 mins. Pellet cellular debris was collected by centrifugation at 10,000 g for 10 min at 4 °C. The supernatant (protein lysate) was transferred to a fresh 1.5 mL microcentrifuge tube. 1–10 µL primary antibody or the appropriate control IgG (Antibodies used for Western blotting were described in the Reagents and Antibodies part) were added into the protein lysate and incubated at 4 °C for 1 h. 20 µL Protein A/G PLUS-Agarose (cat. sc-2003, Santa Cruz) was resuspended and added into the protein lysate, and then incubated at 4 °C overnight. Immunoprecipitates were centrifugated at 2500 rpm for 5 mins at 4 °C. The Protein A/G PLUS-Agarose was washed 4 times with ice-cold PBS. And then, the pellet was resuspended with 1× sample buffer and boiled for 5 mins. Boiled samples were performed with SDS-PAGE electrophoresis and further immunoblot analysis.

### Chromatin-immunoprecipitation (ChIP) assay

ChIP assay was performed with lysates prepared from melanoma cells after indicated treatment and then measured by SimpleChIP Kit (56383, CST, Danvers, MA, USA) according to the manufacturer’s instructions. Briefly, immunoprecipitation was performed with primary antibody and isotype control IgG antibody (Antibodies used for ChIP were described in Reagents and Antibodies part). The immunoprecipitated DNA was treated with RNase A and proteinase K, followed by purification using phenol-chloroform extraction and ethanol precipitation. The purified DNA and input DNA were analyzed by quantitative real-time PCR. Primer sequences used in this study are listed as follows: *IRE1α*-Forward: 5′- AGTAGCTGGGACTACAGGTGTGC-3′, Reverse: 5′- TCGCTGCTGCTGCTTCTTGAAC′; *SIRT7*- Forward: 5′- AACCCAGGTGATTTCTGTTCTCAGG-3′, Reverse: 5′- GGAGTGCCTCATTCCAACCTTCTG-3′; *CD274*- Forward: 5′- TCAGAGGGCATTGCAGATAGTAGA -3′, Reverse: 5′- GAGCAATTTTGGTGACTGTAAGTTTGG -3′. % Input was calculated as follows: 100% × 2^(–△CT)^, and △CT = Ct (Ip) – Ct (Input).

### Tumor cell killing assay

The co-culture system for T cell and tumor cell was established as described previously.^49^ To acquire activated T cells, human peripheral blood mononuclear cells (PBMCs) were cultured in RPMI 1640 with ImmunoCult Human CD3/CD28/CD2 T cell activator (Cat.10970; STEMCELL Technologies) and Recombinant Human IL-2 (1000 U/mL, 202-1L-050, R&D) for four days. For the construction of the in vitro co-culture system, cancer cells after different treatment according to the purpose of each experiment were incubated for 24 h with previously activated T cells at the ratio of 1:3 with 100 ng/mL anti-CD3 antibody (16-0037; eBioscience) and 1000 U/mL IL-2 (200-02, Peprotech). The living cancer cells were stained with crystal violet followed by quantification at OD 570 nm. T cells were collected and assessed by flow cytometry. Zombie dye (cat. 423108, Biolegend) was used for live/dead cell determination. For surface staining, cells were washed and stained with APC anti-human CD3 antibody, PE-cy7 anti-human CD8a antibody and FITC anti-human CD69 antibody. For GZMB staining, cells were fixed and permeabilized by Transcription Factor Buffer Set (cat. 424401, Biolegend), and then stained with PE- anti-human/ mouse Granzyme B antibody according to the manufactures’ instructions. Stained cells were runed on BD LSRFortessa, and all data were analyzed using FlowJo v10 software (Treestar Inc) and GraphPad Prism 8.0 (GraphPad Software).

### Animal experiments

Animal experiments were approved by the Animal Care and Use Committee of the animal facility at the Fourth Military Medical University. All BALB/c nude mice and C57BL/6 mice used in the experiments were female and approximately 6 weeks old. The tumor volume ([L × W^2^] × 0.5) was calculated based on caliper measurements every 3 days after injection.

In the SIRT7-knockdown experiment, the BALB/c nude mice and C57BL/6 mice were the subcutaneous implantation with 5 × 10^5^ A2058-sh-hNC/sh-hSIRT7-1/sh-hSIRT7-2 melanoma cells, and B16F10-sh-mNC/sh-mSIRT7-1/sh-mSIRT7-2 melanoma cells respectively. After the mice were sacrificed about 19 days later, a part of the tumor was fixed in 4% paraformaldehyde overnight following immunofluorescence staining, and the remaining tumor was prepared for single-cell suspension and analyzed the tumor-infiltrating immune cells by flow cytometry.

In order to evaluate the role of SIRT7 in mouse tumor immune microenvironment under ER stress, 5 × 10^5^ B16F10-sh-mNC melanoma cells or B16F10-sh-mSIRT7-1 melanoma cells were subcutaneously injected into the C57BL/6 mice. When the volume of tumor reached to 50 mm^3^, 700 µg HA15 (ER Stress inducer) was administered following the schedule outlined in Fig. S7a. After the mice was sacrificed, the tumor was prepared for single-cell suspension and analyzed the tumor-infiltrating immune cells via flow cytometry.

To deplete CD8^+^T cells, C57BL/6 mice were injected with 100 μg of anti-CD8α antibody (BioXCell, BE0117, USA) per mouse following the schedule outlined in Fig. 5f as described previously.^68^

To determine whether SIRT7 deficiency can synergize with anti-PD-1 immunotherapy in melanoma, 5 × 10^5^ B16F10-sh-mNC melanoma cells or B16F10-sh-mSIRT7-1 melanoma cells were subcutaneously injected into the C57BL/6 mice. When the tumor volume reached to 50 mm^3^, 100 µg anti-PD-1 antibody (Bio X Cell, BE0146, USA) was administered every 3 days.

### Statistical analysis

Data were presented as the mean ± S.D., of which the statistical analyses were performed using GraphPad Prism (GraphPad Software 8.0; San Diego, CA, USA). Unpaired, two-tailed Student’s *t*-tests was used to analyze the significance between two groups, whereas one-way ANOVA analysis was used to compare the differences among multiple groups. In addition, Pearson correlation analysis was used to evaluate the correlations between measured variables. Except for special descriptions, each experiment was performed at least three times. *P* values of <0.05 were considered statistically significant.

## Supplementary information

Supplementary Materials

## Data Availability

All data were available within the manuscript and supplementary materials, or available from the corresponding author upon reasonable request.
