# Supplementary material for: SIRT7 orchestrates melanoma progression by simultaneously promoting cell survival and immune evasion via UPR activation
Source: Signal Transduct Target Ther. 2023 Mar 15;8:107. doi: 10.1038/s41392-023-01314-w (PMC10015075; doi:10.1038/s41392-023-01314-w)

Supplementary Materials for

SIRT7 orchestrates melanoma progression by simultaneously promoting cell survival and immune evasion via UPR activation

Xiuli Yi^*^, Huina Wang^*^, Yuqi Yang^*^, Hao Wang, Hengxiang Zhang, Sen Guo, Jianru Chen, Juan Du, Yangzi Tian, Jingjing Ma, Baolu Zhang, Lili Wu, Qiong Shi, Tianwen Gao, Weinan Guo^#^, Chunying Li^#^

Correspondence to: lichying@fmmu.edu.cn or guown@fmmu.edu.cn

**This PDF file includes:**

1. Materials and Methods
2. Figures. S1 to S10
3. Tables S1
4. original and uncropped films of Western blots

Materials and Methods

**Materials**

**Antibodies**

For immunoblotting and immunofluorescence staining analysis, following antibodies were used: HSPA5 (cat. 66574-1-Ig, 1:1000 for Immunoblotting (IB), 1:100 for Immunofluorescence (IF), Proteintech), SIRT7 (cat. 5360, 1:1000 for IB, Cell Signaling Technology; cat. 78977, 1:100 for IF, Abcam), GAPDH (cat. 60004-1-Ig, 1:5000, Proteintech), Histone H3 (acetyl K18) (Ac-H3K18, cat. ab1191, 1:1000 for IB, 1:100 for IF, Abcam), -Histone H3 (H3, cat. ab1791, 1:1000 for IB), PARP1 (cat. 13371-1-AP, 1:1000, Proteintech), Cleaved Caspase-3 (cat. 9664, 1:1000 for IB, 1:100 for IF, Cell Signaling Technology), Phospho PERK (Thr980) (cat. 3179, 1:1000 for IB, Cell Signaling Technology), ATF6 (cat. 65880, 1:1000 for IB, Cell Signaling Technology), IRE1α (cat. 3294, 1:1000 for IB, Cell Signaling Technology), Phospho IRE1α (ser724) (cat. NB100-2323, 1:1000 for IB, Novus Biologicals), XBP1s (D2C1F) (cat. 12782, 1:1000 for IB, Cell Signaling Technology), ERK1/2 (cat. 4695, 1:1000 for IB, Cell Signaling Technology), Phospho ERK1/2 (Thr202/Tyr204) (cat. 9101, 1:1000 for IB, Cell Signaling Technology), JNK1/2 (cat. 9252, 1:1000 for IB, Cell Signaling), Phospho JNK1/2 (Thr183/Tyr185) (cat. 9251, 1:1000 for IB, Cell Signaling Technology), p38 MAPK (cat. 8690, 1:1000 for IB, Cell Signaling Technology), Phospho p38 MAPK (Thr180/Tyr182) (cat. 4511, 1:1000 for IB, Cell Signaling Technology), Ubiquitin (linkage-specific K48) (cat. 140601, 1:1000 for IB, Abcam), Ubiquitin (linkage-specific K63) (cat. 179434, 1:1000 for IB, Abcam), SMAD4 (cat. 10231-1-AP, 1:1000 for IB, 1:200 for IF, Proteintech), PD-L1(cat. 13684, 1:1000 for IB, Cell Signaling Technology), NF-κB p65 (cat. 8242, 1:1000 for IB, Cell Signaling Technology), Phospho-NF-κB p65 (Ser536) (cat. 3033, 1:1000 for IB, Cell Signaling Technology).

For Co-IP assay, following antibodies were used: SIRT7 (cat. 5360, 1:200, Cell Signaling Technology), SMAD4 (cat. sc-7966, 2 µg per 500 µg of total protein, Santa Cruz), MEF2D (cat. 610774, 1:200, BD Biosciences), Normal Rabbit IgG (cat. 2729, 2 µg per 500 µg of total protein, Cell Signaling Technology), Mouse (G3A1) mAb IgG1 Isotype Control (cat. 5415, 2 µg per 500 µg of total protein, Cell Signaling Technology).

For TMA assay, following antibodies were used: SIRT7 (cat. 78977, 1:100, Abcam), Histone H3 (acetyl K18) (Ac-H3K18, cat. ab1191, 1:100, Abcam), SMAD4 (cat. sc-7966, 1:50, Santa Cruz), XBP1s (cat. NBP1-77681, 1:100, Novus Biologicals), NF-kB p65 (phospho S536) (cat. 86299, 1:100, Abcam), Anti-CD8α antibody (cat. ZA-0508, ZSGB-BIO), Anti-PD-L1 antibody (cat. ab233482, Abcam).

For ChIP assay, following antibodies were used: SMAD4 (cat. sc-7966, Santa Cruz), NF-κB p65 (cat. 8242, Cell Signaling Technology), XBP1s (cat. 40435, Cell Signaling Technology), Normal Rabbit IgG (cat. 2729, Cell Signaling Technology), Mouse (G3A1) mAb IgG1 Isotype Control (cat. 5415, Cell Signaling Technology).

For analysis of tumor-infiltrating immune cells by flow cytometry, following antibodies were used: APC anti-human CD3 (cat. 300312, Biolegend, 5 µL per 10^6^ cells), PE-cy7 anti-human CD8α (cat. 344750, Biolegend, 0.25 µg per 10^6^ cells), FITC anti-human CD69 Antibody (cat. 310904, Biolegend, 5 µL per 10^6^ cells), anti-mouse Fc blocking Ab (cat.101320, Biolegend, 1.0 µg per 10^6^ cells), Pacific Blue anti-mouse CD45 (cat. 103126, Biolegend, 0.25 µg per 10^6^ cells), APC anti-mouse CD3 (cat. 100236, Biolegend, 0.5 µg per 10^6^ cells), PE anti-mouse CD4 (cat. 100408, Biolegend, 0.25 µg per 10^6^ cells), PerCP anti-mouse CD8α (cat. 100732, Biolegend, 0.25 µg per 10^6^ cells), PE-cy7 anti-mouse CD8α (cat. 100722, Biolegend, 0.25 µg per 10^6^ cells), PE-anti-human/ mouse Granzyme B (cat.372208, Biolegend, 1.0 µg per 10^6^ cells), PE-conjugated anti-mouse/human CD11b (cat. 101207, Biolegend, 0.25 µg per 10^6^ cells), APC anti-mouse Ly-6C (cat. 128016, Biolegend, 0.25 µg per 10^6^ cells), PE-cy7 anti-mouse Ly-6G (cat. 127618, Biolegend, 0.25 µg per 10^6^ cells), Brilliant Violet 650™ anti-mouse CD11c (cat. 127618, Biolegend, 0.25 µg per 10^6^ cells) and APC anti-mouse F4/80 (cat. 123116, Biolegend, 0.25 µg per 10^6^ cells).

**Methods**

**Plasmid vectors and siRNA transfection**

Plasmid and siRNA transfection were performed with Lipofectamine 3000 transfection Reagent kit (L3000-015, Invitrogen, Carlsbad, USA) according to the manufacturer’s recommended procedures. Specific custom siRNAs and SIRT7 overexpression plasmid (His-Tagged *SIRT7*) as well as empty plasmid vectors (pCMV3-His) were designed and synthesized in GenePharma (Shanghai, China) and Sino Biological (Beijing, China) respectively. The sequences of used siRNAs against *SIRT7*, *SMAD4*, *XBP1*, *IRE1α* and *MEF2D* were as follows: si-*SIRT7*-1: 5‘- CCCUGAAGCUACAUGGGAAdTdT -3’; si-*SIRT7*-2: 5‘-CCUUUCUGUGAGAACGGAATT-3’; si-*SMAD4*-1: 5‘- GGUGGAGAGAGUGAAACAUTT -3; si-*SMAD4*-2: 5‘- CCAGCAUCCACCAAGUAAUTT -3; si-*XBP1*: 5‘- CUGUCUGUACUUCAUUCAATT -3’; si-*IRE1α*: 5‘- CAUCAACCUCUCUUCUGUATT -3’; si-*MEF2D*: 5‘- GCGAGAUCGCACUCAUCAUCU -3’; si-NC: 5‘- UUCUCCGAACGUGUCACGUTT-3.

**NAD^+^ and NADH level examination**

NAD^+^ and NADH level was accessed using the NAD^+^/NADH Assay Kit with WST-8 (cat. S0175, Beyotime, Shanghai, China) according to the manufacturer’s recommendations. Briefly, cells after indicated treatment were collected and washed twice with ice-cold PBS. Cells were lysed with NAD^+^/NADH extract. The supernatant was collected and the NAD_total_ was detected. To detect the concentration of NADH in the sample, 100 μL of the supernatant was heated in PCR instrument at 60 ºC for 30 minutes to decompose NAD^+^. Alcohol dehydrogenase working solution was added to each sample before mixing, and then processed to incubation for 10 mins at 37 °C. The chromogen solution was added to each sample before mixing, and then processed to incubation for 30 mins at 37 ºC. The output was measured immediately on a colorimetric microplate reader at optical density of 450 nm (Bio-Rad, Hercules, CA, USA). NAD^+^ = NAD_total_- NADH, NAD^+^/NADH = (NAD_total_- NADH)/NADH.

**RNA extraction and quantitative real-time PCR**

Total RNA was extracted using TRIzol reagent (cat. 15596018, Invitrogen, Carlsbad, CA, USA). mRNA was reversely transcribed to cDNA using the PrimeScript^TM^ RT Master Mix kit (cat. RR036A, TaKaRa, Tokyo, Japan), and qRT-PCR was performed using SYBR Premix Ex TaqTM II kit (cat. RR820A, TaKaRa, Tokyo, Japan) with a BIO-RAD Multicolor Real-time PCR Detection System (iQTM5, Bio-Rad, Hercules, CA, USA). The primers were listed in Supplementary Table S1. Relative quantification was performed according to the ΔΔCT method, and results were expressed in the linear form using the formula 2-^ΔΔCT^. β-actin mRNA was used as an internal control.

**Immunoblotting analysis**

For Immunoblotting analysis, cells after indicated treatment were washed with phosphate-buffered saline (PBS) and lysed with cell lysis solution (RIPA, Beyotime, Shanghai, China) supplemented with protease and phosphatase inhibitor cocktail (cat. 5872s, Cell Signaling Technology), and then total protein extracts were obtained and quantified using BCA Protein Assay kit (Beyotime, Shanghai, China). Equal amounts of protein were separated by SDS polyacrylamide gel electrophoresis (PAGE) and then transferred onto polyvinylidene fluoride membranes (Millipore, Billerica, MA). After blocking in a solution of 6% non-fat dry milk diluted in Tris-buffered saline, the membranes were washed and incubated with primary antibodies overnight at 4 °C. Antibodies used for western blotting were described in **Reagents and Antibodies** part. After being washed, the membranes were incubated with horseradish peroxidase-conjugated secondary antibodies for 2 h at room temperature. Bound antibodies were detected using the ECL western blotting detection system (Bio-Rad, Hercules, CA, USA). Image J software was used to analyze and quantify the bands of western blotting. GraphPad Prism (GraphPad Software 8.0, San Diego, CA, USA) was used to analyze the correlation between the expression of SIRT7 relative to GAPDH and Ac-H3K18 relative to H3.

**Immunofluorescence staining analysis**

For Immunofluorescence staining of frozen tissue sections, the tissue sections were blocked with goat serum (Gibco, USA) for 30 mins and then incubated with primary antibodies overnight at 4 °C, followed by 1 h incubation with appropriate secondary antibodies (cat. EK022, goat anti-rabbit IgG H&L (Cy3), 1:100; cat. EK041, goat anti-rat IgG H&L (FITC), 1:100 and cat. EK013, goat anti-mouse IgG H&L (FITC), 1:100, Zhuangzhibio, Xi’an, China). Hoechst 33258 (cat. C1011, Beyotime, Shanghai, China) was used as a counterstain.

For Immunofluorescence staining of paraffin-embedded tissue sections, tissue sections were deparaffinized and rehydrated with graded ethanol dilutions. After antigen retrieval in Tris-EDTA buffer (10 mM, pH 9.0), the remaining steps were the same as that for immunofluorescence staining of frozen tissue sections.

For Immunofluorescence staining of cultured cells, cells on coverslips were fixed in 4% paraformaldehyde for 10 mins, permeabilized with 0.5% Triton X-100 for 10 mins, and blocked with goat serum (Gibco, USA) for 30 mins. The cells were then incubated with primary antibodies overnight at 4 °C, followed by secondary antibodies for 1 h at room temperature. Antibody information was listed in in **Reagents and Antibodies** part. Images were acquired by a Zeiss LSM880 confocal laser scanning microscope (Zeiss, German). All experiments were independently repeated at least three times.

**Immunohistochemical staining analysis of tumor tissue microarray**

Melanoma tissue microarray (M1004e) was purchased from US Biomax Inc. Detailed information can be accessed via <http://www.biomax.us/tissue-arrays/Melanoma/ME1004e>.

Melanoma tissue microarray (K063Me01) was purchased from Bioaitech Inc. Detailed information can be accessed via <https://www.bioaitech.com/chip> design/7c63645dbaef4b6aac51daa5db3c96fe.html.

For immunohistochemical staining analysis, paraffin-embedded melanoma tissues of tumor tissue microarray (TMA) were de-paraffinized and rehydrated with graded ethanol dilutions. After antigen retrieval in Tris-EDTA Buffer (10 mM Tris Base, 1 mM EDTA Solution, 0.05% Tween 20, pH 9.0), goat serum was added to block nonspecific binding for 30 mins. Tissue sections were then incubated with antibodies at 4 °C overnight, followed by horseradish peroxidase-conjugated goat anti-rabbit/mouse IgG (ComWin Biotech, Beijing, China) for 40 minutes at room temperature. Subsequently, the bounded antibody was detected using 3-amino-9- ethylcarbazole (AEC, SOLARBIO SCIENCE & TECHNOLOGY, Beijing, China), and the sections were counterstained with hematoxylin and mounted with glycerol. The staining scores were evaluated as described previously.^1^ Briefly, the percentages of staining-positive cells were scored into four categories: 0 (0%), 1 (1-33%), 2 (34-66%), and 3 (67-100%). The staining intensities were scored into four grades: 0 (none), 1 (weak), 2 (moderate), and 3 (strong). The final staining score was defined as the product of the percentage and the intensity scores.

**Cell viability assay**

Cell viability was examined by the cell counting kit-8 (CCK-8) assay (7seabiotech, Shanghai, China) following the manufacturer’s instructions. In brief, cells were seeded in 96-well plates (5000 cells per well) and treated as previously described. After treatment, the cultured medium was replaced by the CCK-8 reagent diluted 1:10 in fresh medium and incubated for 1 h at 37 °C. The absorbance at 450 nm was measured using a Model 680 Microplate Reader (Bio-Rad, Hercules, CA, USA).

**Colony formation assay**

For colony formation assay, cells were collected, counted, and replated in appropriate dilutions in six-well plates with indicated treatment. After 10-14 days of incubation, colonies were washed with PBS, fixed with 4% paraformaldehyde for 10-20 mins, and then stained with a mixture of 6% glutaraldehyde and 0.5% crystal violet for 1-2 h. After removal of glutaraldehyde crystal violet mixture carefully, the plates with colonies washed with PBS were leaved to dry naturally at room temperature. The number of clones were counted using Image J software.

**Transwell migration and invasion assay**

For transwell migration assays, A2058 and A375 cells transfected with indicated siRNAs or plasmids were starved in serum-free medium for 12 h, then cells were reseeded on the top chambers of 24-well transwell culture inserts (Corning, NY). For invasion assays, transwell chambers with 8 μm-pore size membrane filter inserts (Corning) were coated with Matrigel (BD Biosciences, NJ). After 24 h, cells were fixed in 4% paraformaldehyde for 10 mins and stained with crystal violet for 30 mins at room temperature. For the quantification of the migratory and invasive cells, Image J software was used to count the stained cells.

**Hypoxia Treatments**

For the induction of hypoxia, melanoma cells were incubated for 24 h to 48 h in a Modulator Incubator Chamber in the hypoxic conditions (1% O_2_). The control cells were cultured in the normoxic incubator (21% O_2_). After treatment, cells were collected and subjected to cell viability assay, RNA extraction and quantitative real-time PCR, immunoblotting analysis and cell apoptosis by flow cytometry, respectively.

**hTF (Transcription factor) target analysis**

Promoter region sequences for human *IRE1α, CD274* and *SIRT7* were obtained via https://www.ncbi.nlm.nih.gov/gene/. The binding sites for transcription factor SMAD4 to *IRE1α* promoter, transcription factor XBP1 to *CD274* promoter and transcription factor NF-κB p65 to *SIRT7* promoter were predicted by JASPAR databases. According to the predicted scores, the higher scoring binding sites (Figure 4b, Figure S9a and Figure S10a) was chosen for validation by Chromatin-immunoprecipitation (Ch-IP) assay, the most significantly enriched sites were used for real time PCR analysis.

**ELISA assay**

ELISA analysis on culture medium of melanoma cells after indicated treatment was performed using the Human IL-8 ELISA Kit (cat. E-EL-H6008, Elabscience), Human TNF-α ELISA Kit (cat. E-EL-H0109, Elabscience) and Human VEGFα ELISA Kit (cat. E-EL-H0111, Elabscience) according to the manufacturer’s instructions. The absorbance (450 nm) was measured with a plate reader (Bio-Rad).

**REFERENCES**

1 Guo, W. et al. ATP-Citrate Lyase Epigenetically Potentiates Oxidative Phosphorylation to Promote Melanoma Growth and Adaptive Resistance to MAPK Inhibition. *Clin Cancer Res*. **26**, 2725-2739 (2020).

**Figure S1. SIRT7 is a stress-responsive factor and significantly up-regulated in melanoma**

**
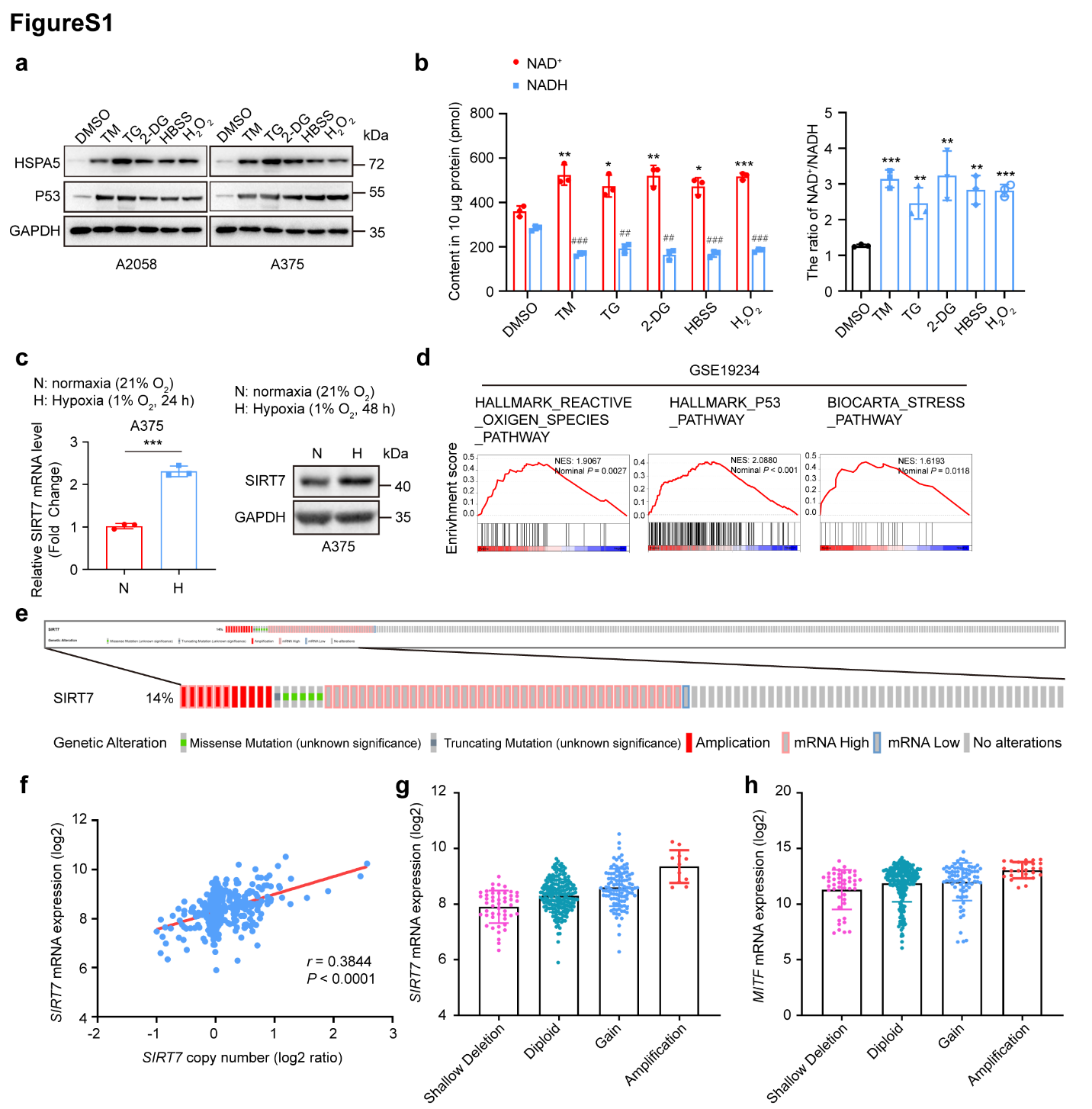
**

**Figure S1 (a)** Immunoblotting analysis of HSPA5 and P53 in A2058 and A375 cells treated with TM (3 μM), TG (1 μM), 2-DG (10 mM), HBSS or H_2_O_2_ (400 μM) for 24 h. **(b)** NAD^+^ and NADH level in A2058 cells treated with TM (3 μM), TG (1 μM), 2-DG (10 mM), HBSS or H_2_O_2_ (400 μM) for 24 h. **(c)** Relative mRNA and protein levels of SIRT7 in A375 cells under normoxic and hypoxic condition. **(d)** GSEA analysis between SIRT7 expression and reactive oxygen species, unfolded protein response, P53 pathway and BIOCARTA stress pathway in GEO dataset (GSE19234). **(e)** The analysis of genomic profile of SIRT7 in TCGA SKCM database. **(f)** The correlation analysis between copy number of *SIRT7* with its mRNA expression in TCGA SKCM database. **(g)** The correlation analysis between ploidy status of *SIRT7* with its mRNA expression in TCGA SKCM database. **(h)** The correlation analysis between ploidy status of *MITF* with its mRNA expression in TCGA SKCM database. *r* value was calculated by Spearman correlation. Data represent the mean ± SD of triplicates. *P* value was calculated by two tailed Student’s t-test. ^*^*P* < 0.05, ^**^*P* < 0.01, ^***^*P* < 0.001, compared to DMSO for NAD^+^ or the ratio of NAD^+^/NADH; ^##^*P* < 0.01, ^###^*P* < 0.001, compared to DMSO for NADH.

**Figure S2. SIRT7 enables melanoma cell survival under stress**

**
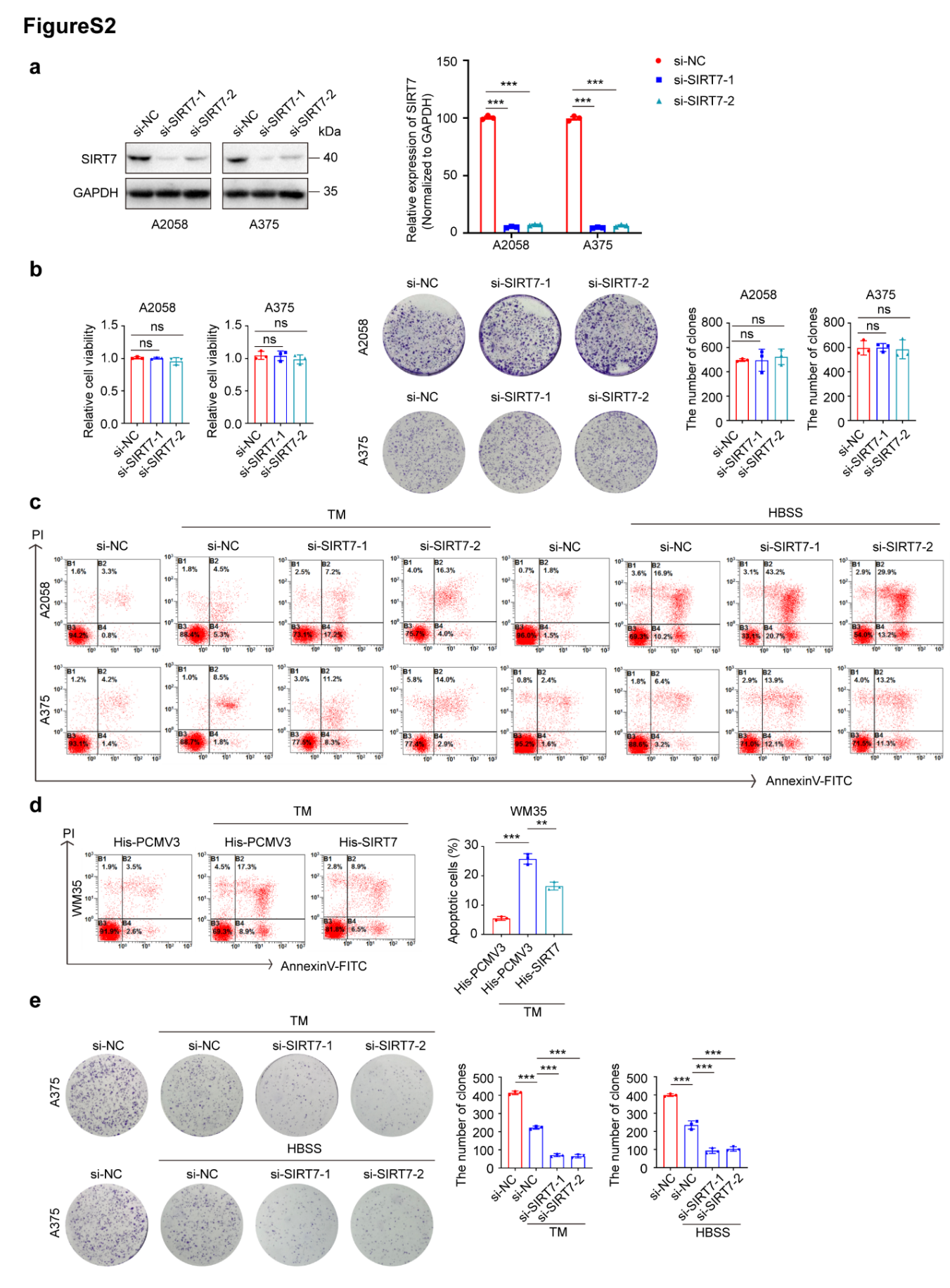
**

**Figure S2 (a)** Immunoblotting analysis the knockdown efficiency of SIRT7 in A2058 and A375 cells. **(b)** Cell viability and colony formation of both A2058 and A375 cells with or without the knockdown of SIRT7. **(c)** Representative images of flow cytometry analysis of cell apoptosis in both A2058 and A375 cells with indicated treatment. **(d)** Flow cytometry analysis of cell apoptosis in WM35 cells with indicated treatment. **(e)** Colony formation analysis of A375 cells with indicated treatment. Data represent the mean ± SD of triplicates. *P* value was calculated by two-tailed Student’s t-test. ^**^*P* < 0.01, ^***^*P* < 0.001. ns, non-significant.

**Figure S3. SIRT7 enables melanoma cell survival under ER stress and hypoxic condition**

**
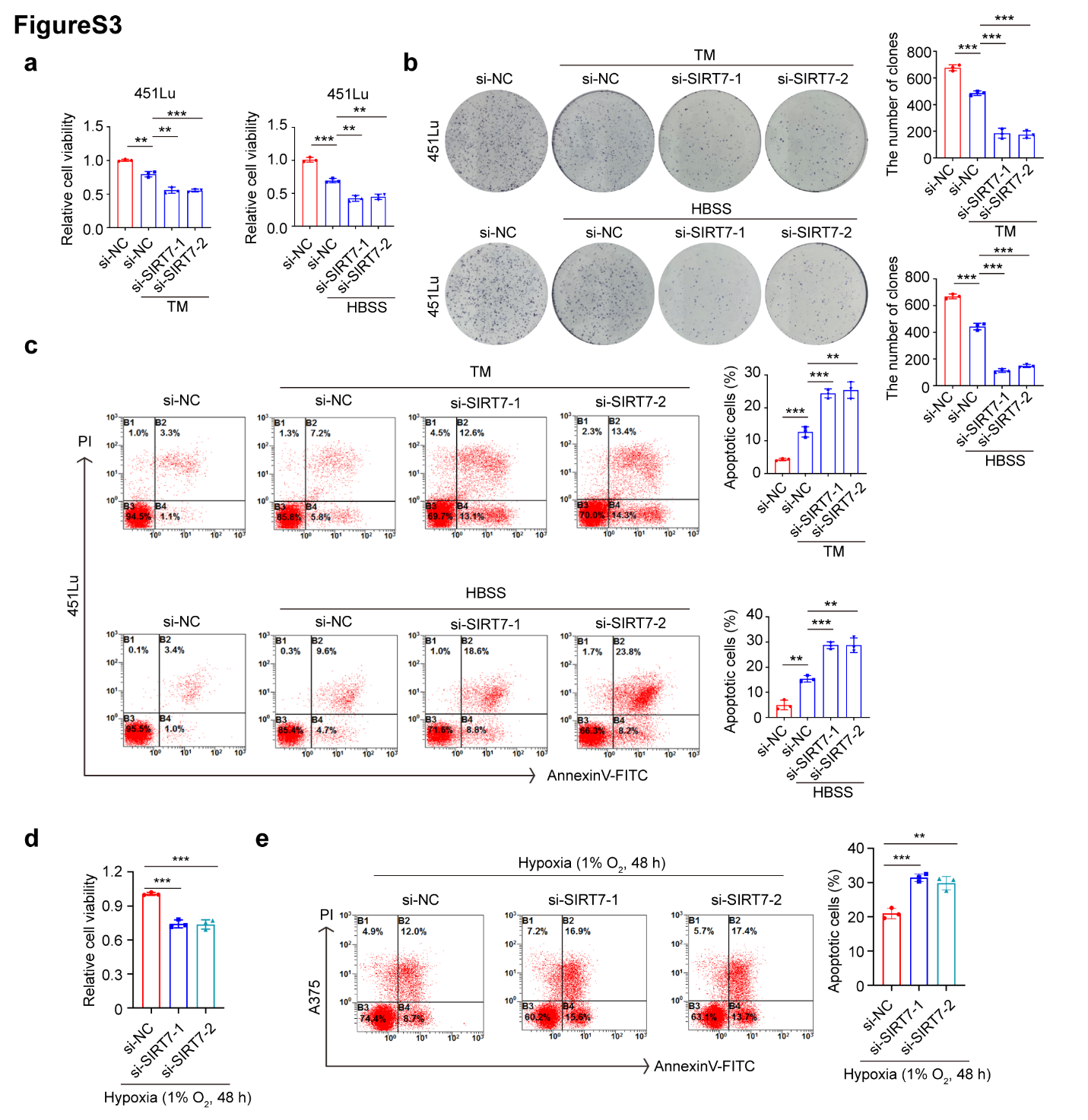
**

**Figure S3 (a)** Cell viability of 451Lu cells treated with TM (3 μM) or HBSS for 24 h with or without the knockdown of SIRT7. **(b)** Colony formation of 451Lu cells treated with TM (3 μM) or HBSS for 24 h with or without the knockdown of SIRT7. **(c)** Cell apoptosis rate of 451Lu cells treated with TM (3 μM) or HBSS for 24 h with or without the knockdown of SIRT7. **(d)** Cell viability of A375 cells under hypoxic condition. **(e)** Cell apoptosis rate of A375 cells under hypoxic condition. Data represent the mean ± SD of triplicates. *P* value was calculated by two-tailed Student’s t-test. ^**^*P* < 0.01, ^***^*P* < 0.001.

**Figure S4** **SIRT7 has little impact on the invasion or migration of melanoma cells**
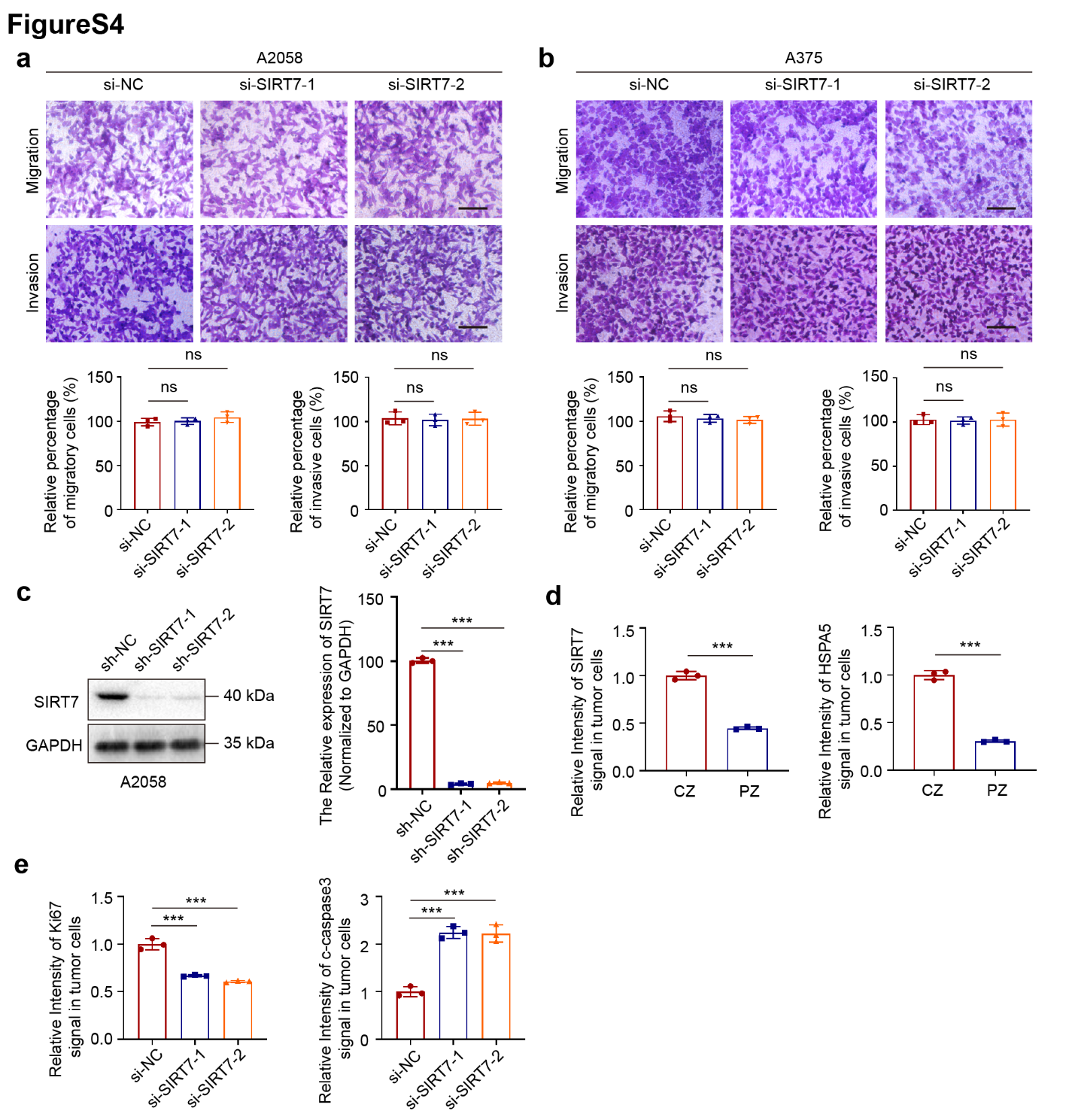


**Figure S4 (a-b)** Invasion and migration assay of both A2058 and A375 melanoma cells with or without the knockdown of SIRT7. Representative fields and quantification of the invaded and migrated cells are shown. Scale bar = 100 μm. **(c)** Immunoblot analysis the knockdown efficiency of SIRT7 in A2058 cells. **(d)** Intensity of SIRT7 and HSPA5 signal in tumors was quantified using Image J software. CZ, central zone; PZ, peripheral zone. **(e)** Intensity of ki67 and cleaved-caspase3 signal in tumors was quantified using Image J software. Data represent the mean ± SD of triplicates. *P* value was calculated by two-tailed Student’s t-test. ^**^*P* < 0.01, ^***^*P* < 0.001. ns, non-significant.

**Figure S5. SIRT7 selectively activates IRE1α-XBP1 branch of UPR and de-acetylates SMAD4 to regulate IRE1α transcription**

**
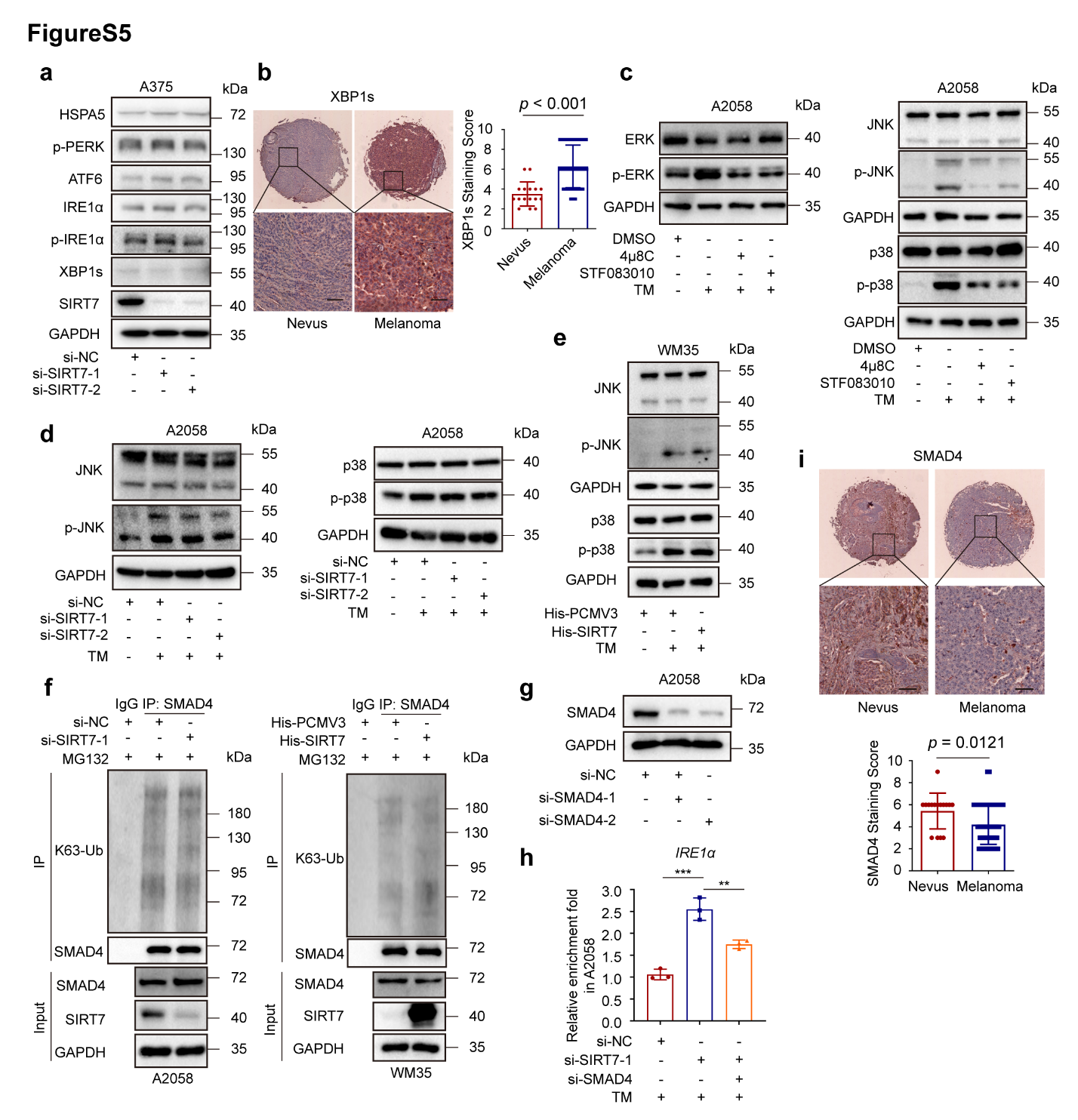
**

**Figure S5 (a)** Immunoblotting analysis of the expressions of three main branches of UPR in A375 cells with indicated treatment. ATF6 (G), Glycosylated-ATF6; ATF6 (N), Non-glycosylated-ATF6. **(b)** Immunohistochemical staining analysis of XBP1s in TMA. Scale bar = 100 μm. **(c)** Immunoblotting analysis of ERK and phosphor-ERK, JNK and phosphor-JNK, and p38 and phosphor-p38 in A2058 cells with indicated treatment. **(d-e)** Immunoblotting analysis of JNK and phosphor-JNK, and p38 and phosphor-p38 in A2058 cells and WM35 cells with indicated treatment. **(f)** Co-immunoprecipitation analysis of interaction between SMAD4 and K63-linked ubiquitin chain in A2058 cells with or without the knockdown of SIRT7 and WM35 cells with or without SIRT7 overexpression treated with MG132. **(g)** Immunoblotting analysis the knockdown efficiency of SMAD4 in A2058 cells. **(h)** Chromatin immunoprecipitation analysis of the enrichment of SMAD4 to the promoter of *IRE1α* in A2058 cells with indicated treatment. **(i)** Immunohistochemical staining analysis of SMAD4 in TMA. Scale bar = 100 μm. Data represent the mean ± SD of triplicates. *P* value was calculated by two-tailed Student’s t-test. ^**^*P* < 0.01, ^***^*P* < 0.001.

**Figure S6. SIRT7 up-regulation eradicates anti-tumor immunity**

**
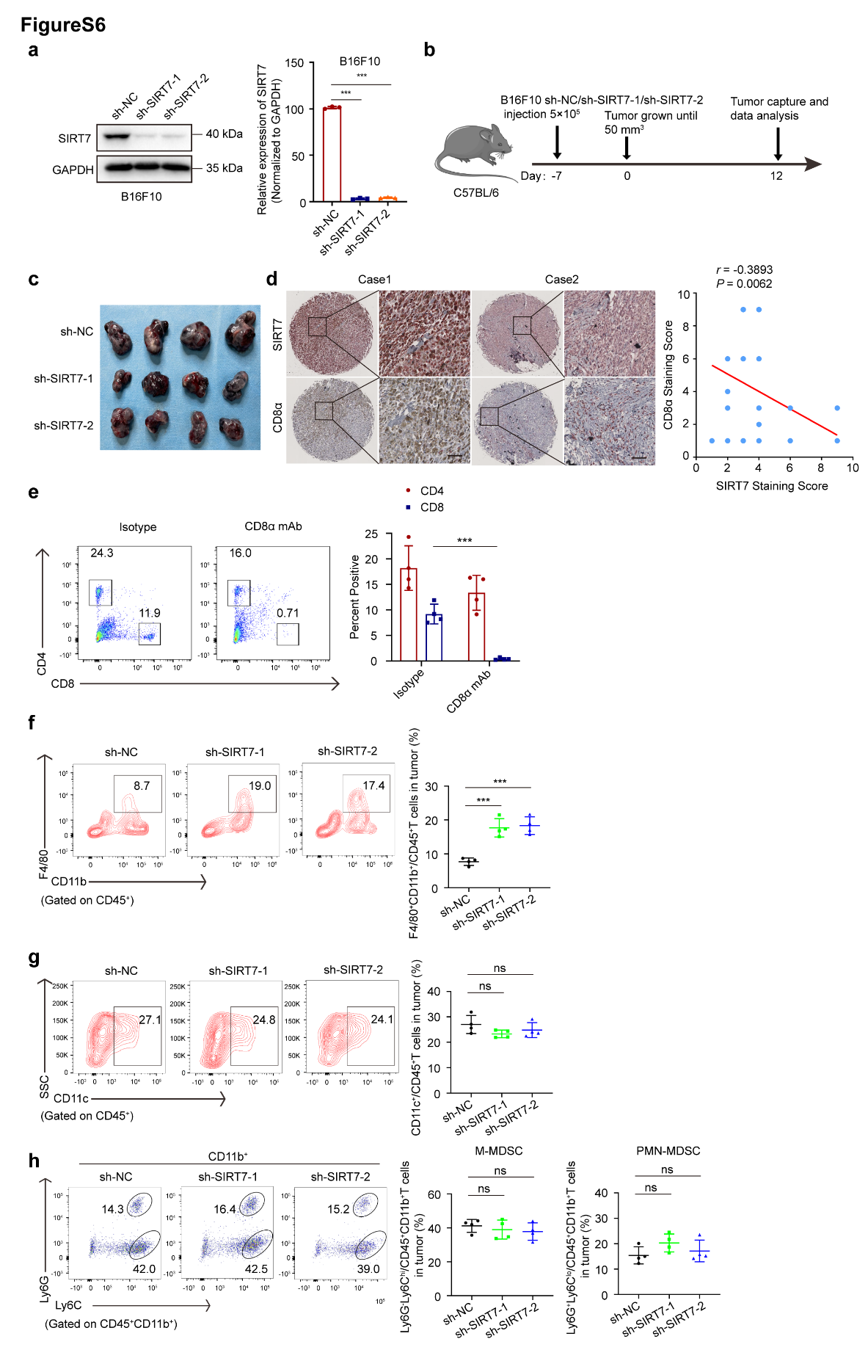
**

**Figure S6 (a)** Immunoblotting analysis the knockdown efficiency of SIRT7 in B16F10 cells. Data represent the mean ± SD of triplicates. **(b)** A schematic view of the treatment plan that C57BL/6 mice burdened with negative control (sh-NC) and two independent SIRT7 shRNA Lentivirus (sh-SIRT7-1 and sh-SIRT7-2)-transfected B16F10 cells. **(c)** images of isolated tumors from mice that received indicated treatment. **(d)** Immunohistochemical staining and correlation analysis of SIRT7 and CD8α in TMA. Scale bar = 100 μm. *r* value was calculated by Spearman correlation*.* **(e)** Representative flow cytometry and quantification of CD4 and CD8α staining of peripheral blood to confirm depletion. **(f)** FACS of CD11b^+^ F4/80^+^ macrophages in CD45^+^cells from B16F10 xenografts with indicated treatment and corresponding quantification. **(g)** FACS of CD11c^+^ DCs in CD45^+^cells from B16F10 xenografts with indicated treatment and corresponding quantification. **(h)** FACS of M-MDSCs (Ly6G^-^Ly6C^hi^ in CD45^+^CD11b^+^ cells) and PMN-MDSCs (Ly6G^+^Ly6C^lo^ in CD45^+^CD11b^+^ cells) from B16F10 xenografts with indicated treatment and corresponding quantification. Symbols of one dot indicates one mouse, and the error bars are mean with ± S.D. *P*-value was calculated by two-tailed Student’s t-test. ^***^*P* < 0.001, ns, non-significant.

**Figure S7. SIRT7 up-regulation eradicates anti-tumor immunity under ER stress**


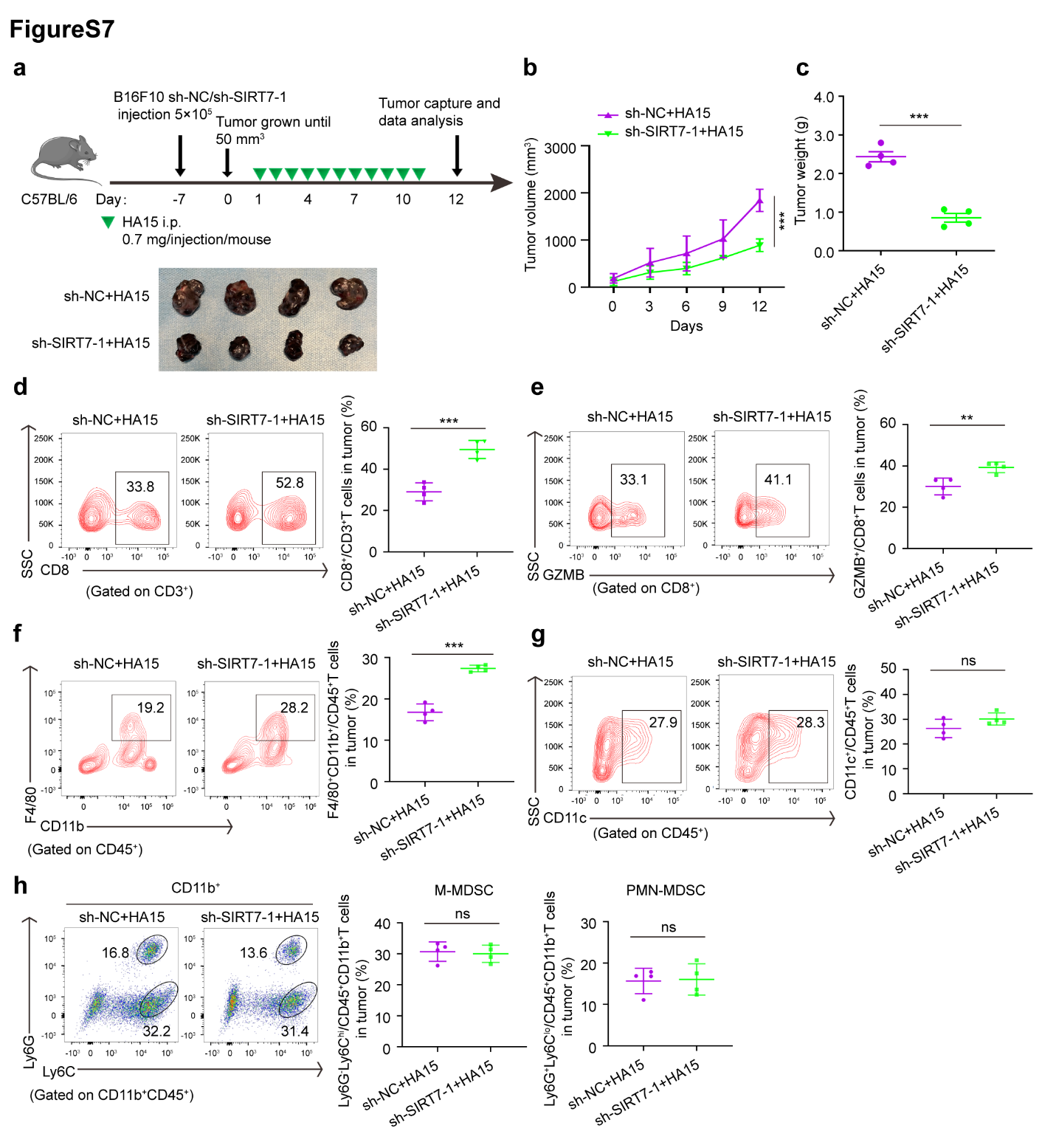


**Figure S7 (a)** A schematic view of the treatment plan that C57BL/6 mice burdened with negative control (sh-NC) and sh-SIRT7-1-transfected B16F10 tumors with HA15 treatment as indicated. Images of isolated tumors from mice that received indicated treatment. Tumor volumes and weights in each group were calculated and displayed in **(b)** and **(c)**. The quantification of FACS of CD8^+^ in CD3^+^ TILs **(d)** and GZMB^+^CD8^+^ TILs **(e)** from B16F10 xenografts with indicated treatment. **(f)** FACS of CD11b^+^ F4/80^+^ macrophages in CD45^+^cells from B16F10 xenografts with indicated treatment and corresponding quantification. **(g)** FACS of CD11c^+^ DCs in CD45^+^cells from B16F10 xenografts with indicated treatment and corresponding quantification. **(h)** FACS of M-MDSCs (Ly6G^-^Ly6C^hi^ in CD45^+^CD11b^+^ cells) and PMN-MDSCs (Ly6G^+^Ly6C^lo^ in CD45^+^CD11b^+^ cells) from B16F10 xenografts with indicated treatment and corresponding quantification. Symbols of one dot indicates one mouse, and the error bars are mean with ± S.D. *P*-value was calculated by two-tailed Student’s t-test. ^**^*P* < 0.01, ^***^*P* < 0.001, ns, non-significant.

**Figure S8.** **SIRT7 up-regulation compromises anti-tumor immunity by promoting PD-L1**


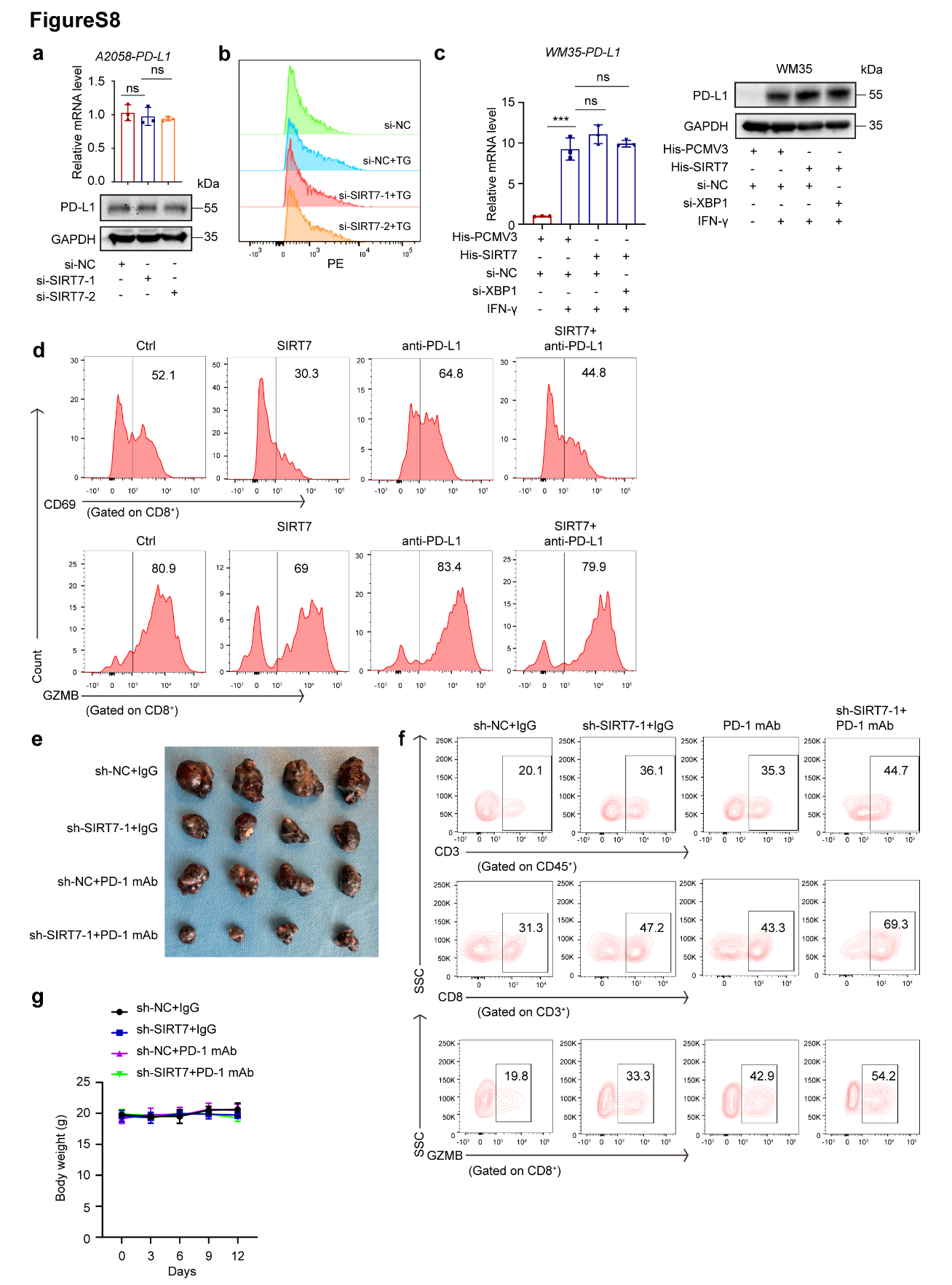


**Figure S8 (a)** Relative mRNA and protein levels of PD-L1 in A2058 cells with indicated treatment. Data represent the mean ± SD of triplicates. **(b)** Representative images of membrane expression of PD-L1 in A2058 cells with indicated treatment detected by flow cytometry. **(c)** Relative mRNA and immunoblotting analysis of PD-L1 in WM35 cells with indicated treatment. Data represent the mean ± SD of triplicates. **(d)** Representative images of CD69^+^in CD8^+^T cells and GZMB^+^CD8^+^ TILs from co-culture system with indicated treatment detected by flow cytometry. **(e)** Images of isolated tumors from mice that received indicated treatment. **(f)** The FACS of CD3^+^in CD45^+^cells, CD8^+^ in CD3^+^ TILs and GZMB^+^CD8^+^ TILs from negative control (sh-NC) and sh-SIRT7-1-transfected B16F10 xenografts with or without PD-1 mAb treatment as indicated. **(g)** Body weight of mice that received indicated treatment. Symbols of one dot indicates one mouse, and the error bars are mean with ± S.D. *P*-value was calculated by two-tailed Student’s t-test. ^***^*P* < 0.001, ns, non-significant.

**Figure S9.** **SIRT7 promotes PD-L1 expression via IRE1α-XBP1 axis**


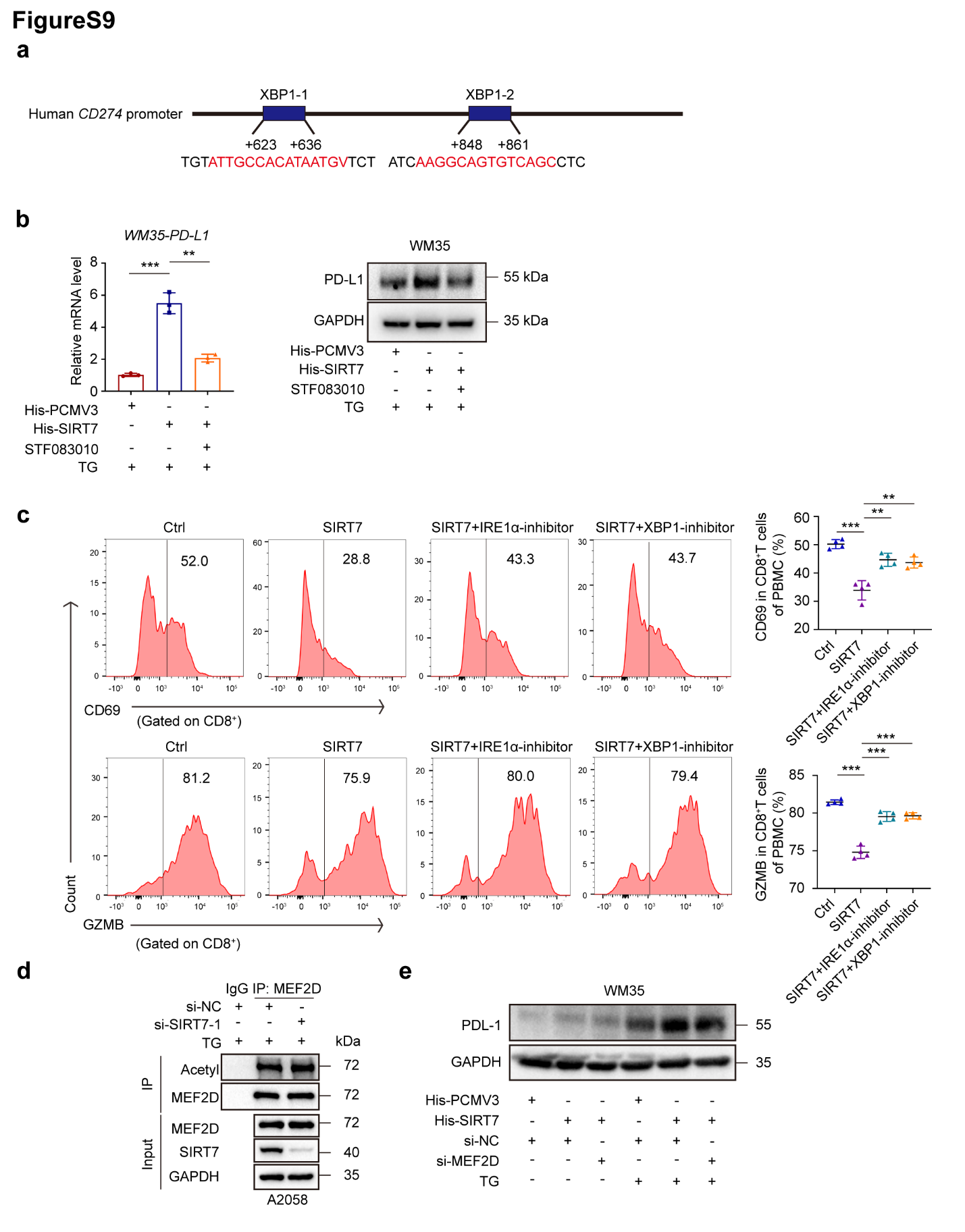


**Figure S9 (a)** Transcription factor analysis of *CD274* promoter identified three XBP1 binding sites. **(b)** Relative mRNA and immunoblotting analysis of PD-L1 in WM35 cells with indicated treatment. **(c)** FACS of CD69^+^in CD8^+^T cells and GZMB^+^CD8^+^ TILs from co-culture system with indicated treatment and corresponding quantification. WM35 cells transfected with control or SIRT7 overexpression plasmid were treated with TG, IRE1α inhibitor (STF083010) or XBP1 inhibitor (Toyocamycin), and then co-cultured with activated T cells for 24 h. Symbols of one dot indicates one PBMC sample. **(d)** Co-immunoprecipitation analysis of acetylation level of MEF2D with indicated treatment in A2058 cells. **(e)** Immunoblotting analysis of PD-L1 in WM35 cells with indicated treatment. Data represent the mean ± SD of triplicates. *P*-value was calculated by two-tailed Student’s t-test. ^**^*P* < 0.01, ^***^*P* < 0.001.

**Figure S10. NF-κB p65 contributes to SIRT7 up-regulation in melanoma**

**
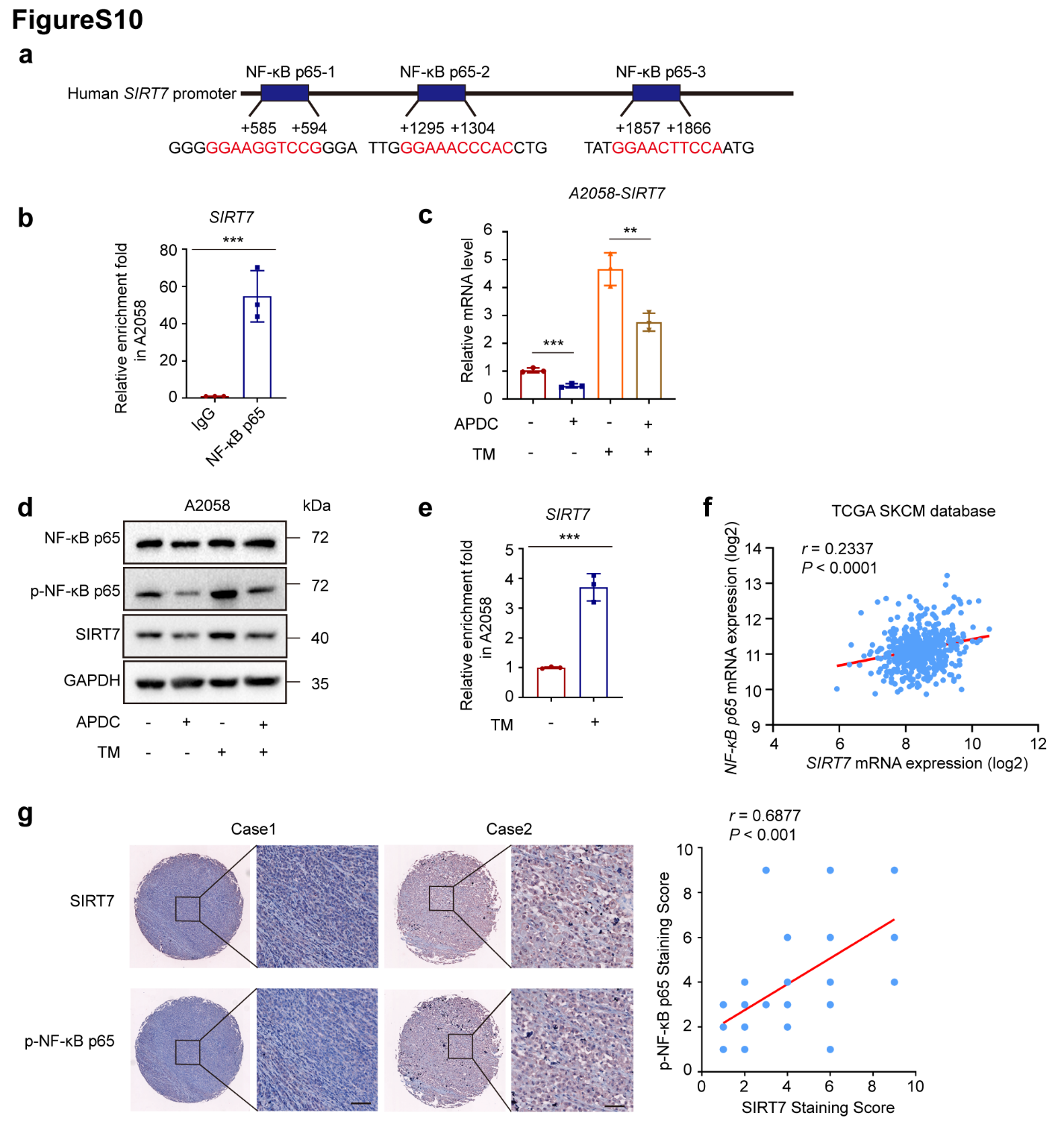
**

**Figure S10 (a)** Transcription factor analysis of *SIRT7* promoter identified three NF-κB p65 binding sites. **(b)** Chromatin immunoprecipitation analysis of the enrichment of NF-κB p65 to the promoter of SIRT7 in A2058 cells. **(c)** Relative mRNA level of *SIRT7* in A2058 cells treated with TM (3 μM) and APDC (10 μM) for 24 h. **(d)** Immunoblotting analysis of NF-κB p65, p-NF-κB p65 and SIRT7 in A2058 cells treated with TM (3 μM) and APDC (10 μM) for 24 h. **(e)** Chromatin immunoprecipitation analysis of the enrichment of NF-κB p65 to the promoter of *SIRT7* in A2058 cells treated with TM (3 μM) for 24 h. **(f)** Correlation analysis of SIRT7 and NF-κB p65 expression in TCGA SKCM database. **(g)** Immunohistochemical staining and correlation analysis of SIRT7 and p-NF-κB p65 in TMA. Scale bar, 100 μm. Data represent the mean ± SD of triplicates. *r* value was calculated by Spearman correlation, *P* value was calculated by two tailed Student’s t-test. ^**^*P* < 0.01, ^***^*P* < 0.001.

**Table S1. List of primers used in qRT-PCR analyses - Related to Experimental Procedures**

| Gene symbols | Forward | Reverse |
| --- | --- | --- |
| *SIRT1* | TAGAGCCTCACATGCAAGCTCTA | GCCAATCATAAGATGTTGCTGAAC |
| *SIRT2* | CCTCGCCTGCTCATCAACA | TCCTCCGAGGCCCATAATC |
| *SIRT3* | CATTCGGGCTGACGTGATG | AACCACATGCAGCAAGAACCT |
| *SIRT4* | CTCGAAAGCCTCCATTGGGT | TGCAAGGATGATCCCACCAC |
| *SIRT5* | GTCATCACCCAGAACATCGA | ACGTGAGGTCGCAGCAAGCC |
| *SIRT6* | CCCGGATCAACGGCTCTATC | GCCTTCACCCTTTTGGGGG |
| *SIRT7* | CGTCCGGAACGCCAAATAC | GACGCTGCCGTGCTGATT |
| *Sec61A1* | TGTCATCTCCCAAATGCTCTCA | ACAGGTAATAGCAAAGGCCAC |
| *ERDJ4* | TCGGCATCAGAGCGCCAAATCA | ACCACTAGTAAAAGCACTGTGTCCAAG |
| *GRP78* | TGACATTGAAGACTTCAAAGCT | CTGCTGTATCCTCTTCACCAGT |
| *p58IPK* | TGTGTTTGGGATGCAGAACTAC | TCTTCAACTTTGACGCAGCTT |
| *TNFA* | TCTCCCCTGGAAAGGACAC | AAGAGGCTGAGGAACAAGCA |
| *IL8* | CACTGCGCCAACACAGAAAT | GCTTGAAGTTTCACTGGCATC |
| *VEGF* | ATCGAGTACATCTTCAAGCCAT | GTGAGGTTTGATCCGCATAATC |
| *IRE1α* | CATCCCCATGCCGAAGTTCA | CTGCTTCTCTCCGGTCAGGA |
| *PDL1* | TGGCATTTGCTGAACGCATTT | TGCAGCCAGGTCTAATTGTTTT |
| *ACTB* | TCATGAAGTGTGACGTGGACATC | CAGGAGGAGCAATGATCTTGATCT |
| *IRE1α-ChIP* | AGTAGCTGGGACTACAGGTGTGC | TCGCTGCTGCTGCTTCTTGAAC |
| *CD274-ChIP* | TCAGAGGGCATTGCAGATAGTAGA | GAGCAATTTTGGTGACTGTAAGTTTGG |
| *SIRT7-ChIP* | AACCCAGGTGATTTCTGTTCTCAGG | GGAGTGCCTCATTCCAACCTTCTG |

original and uncropped films of Western blots


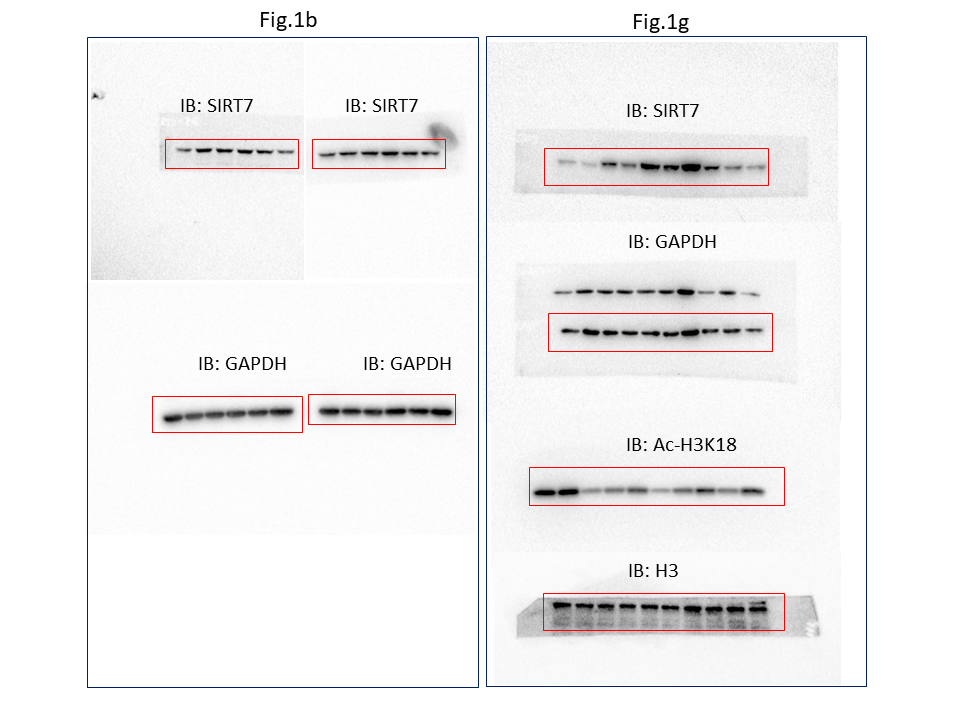


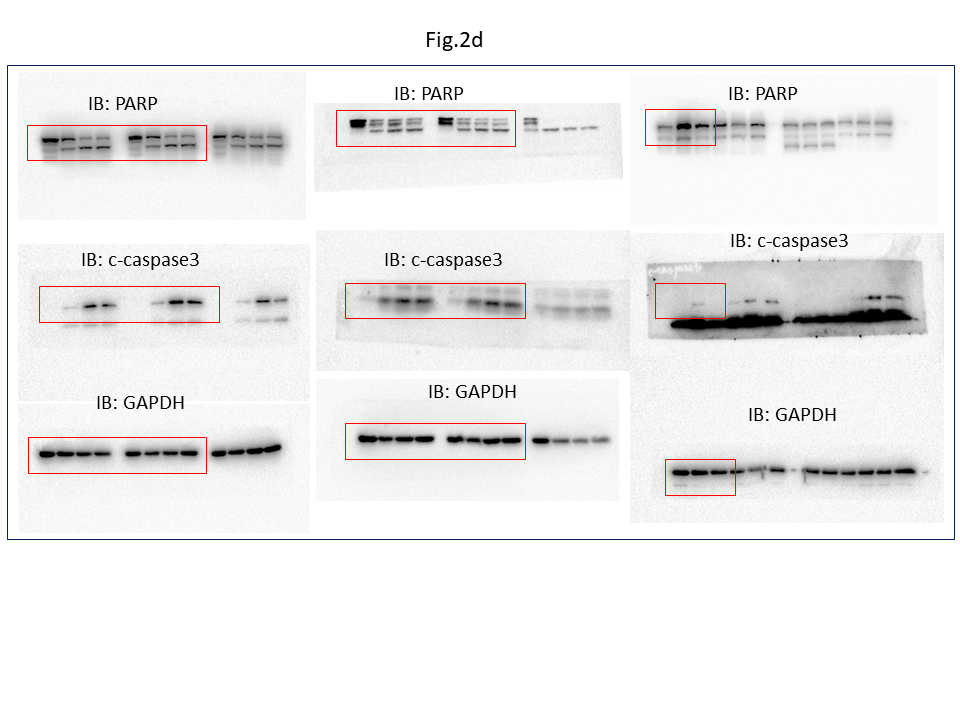

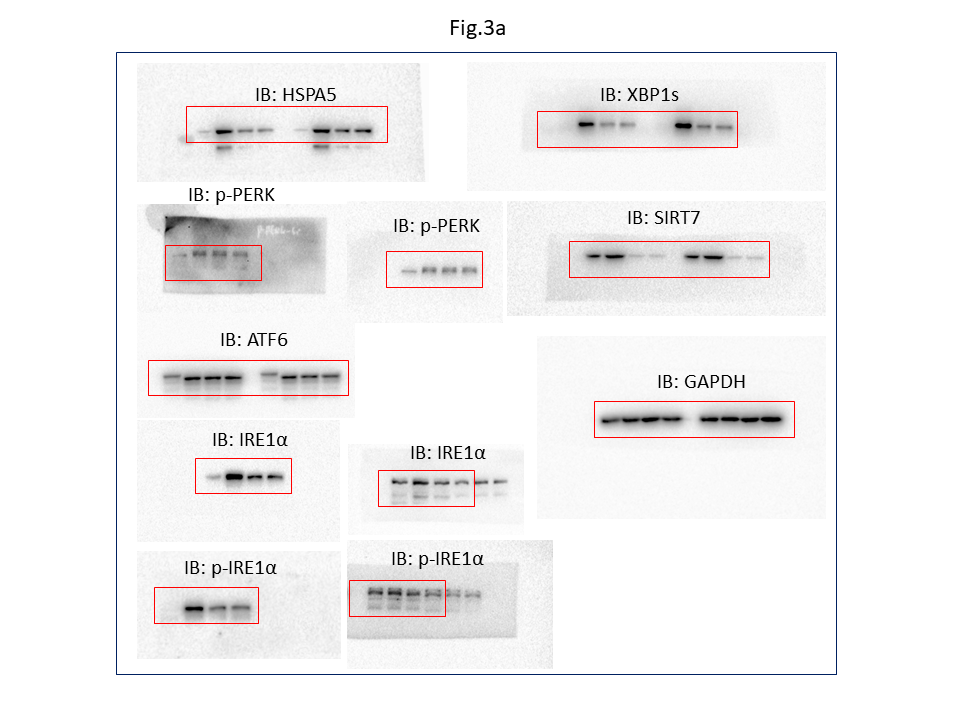


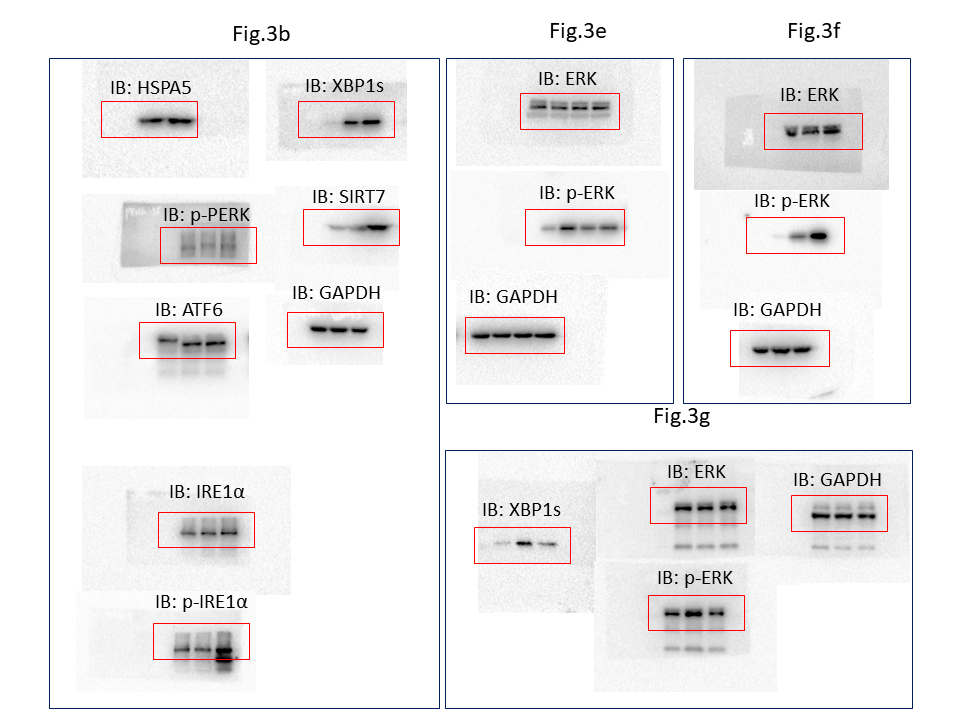


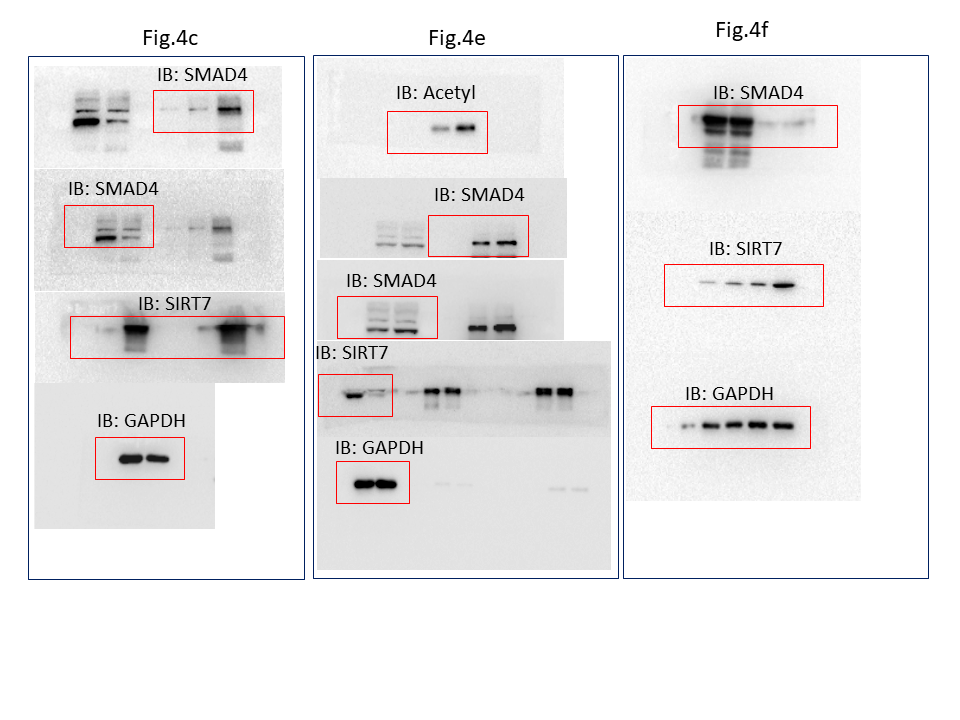


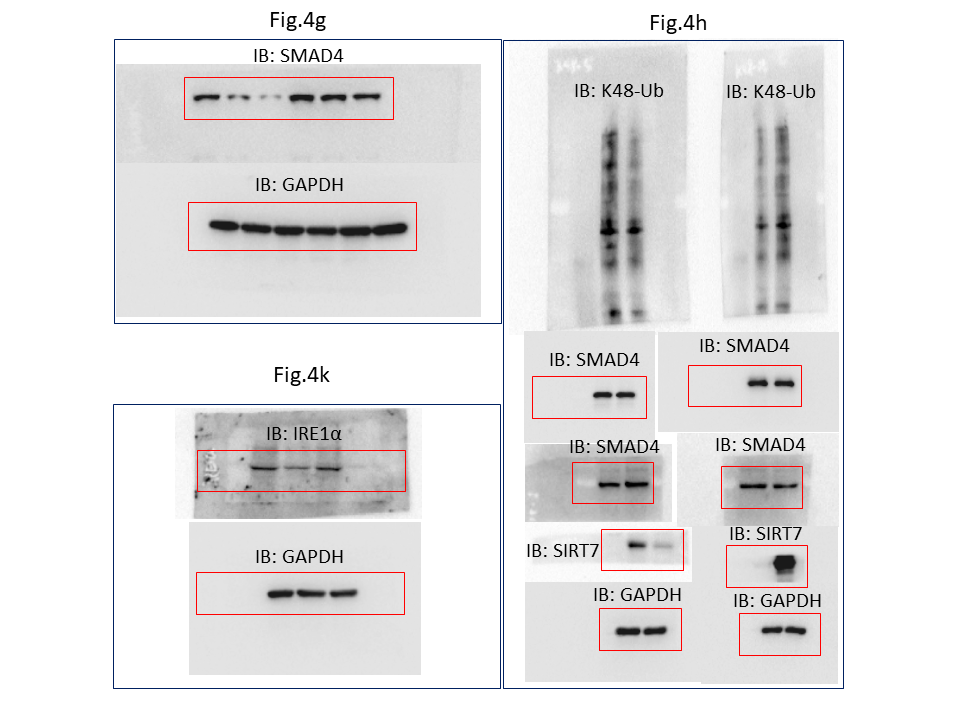


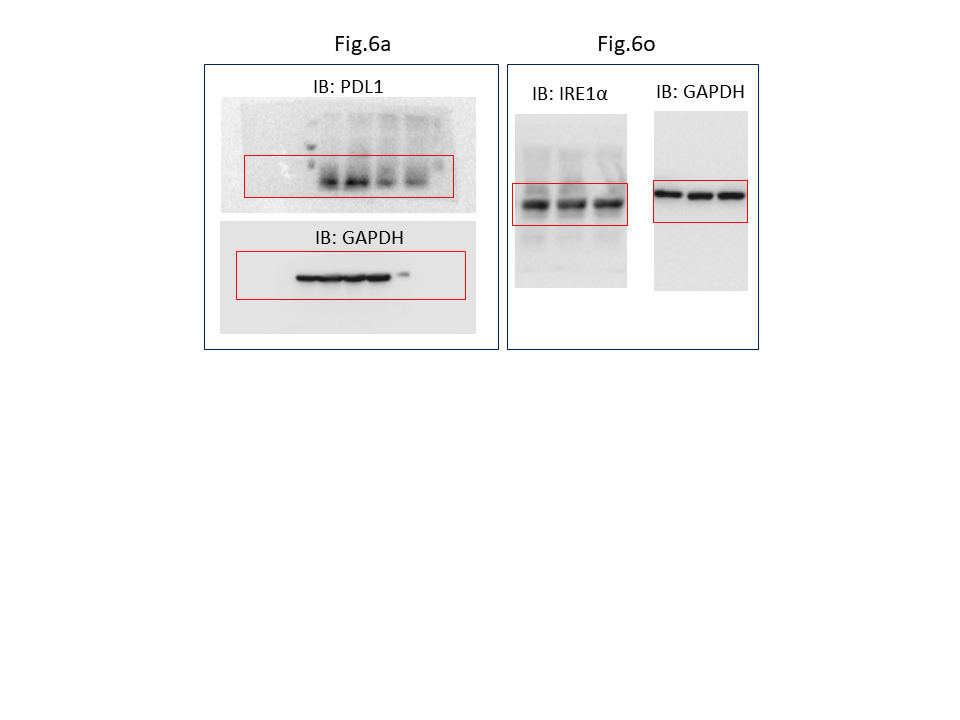


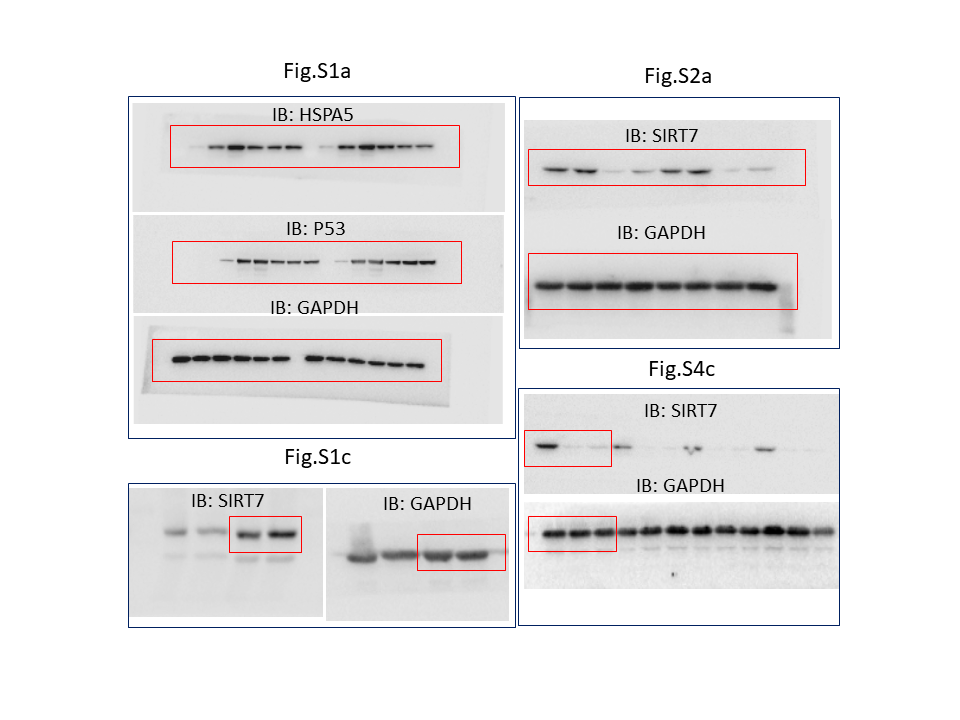


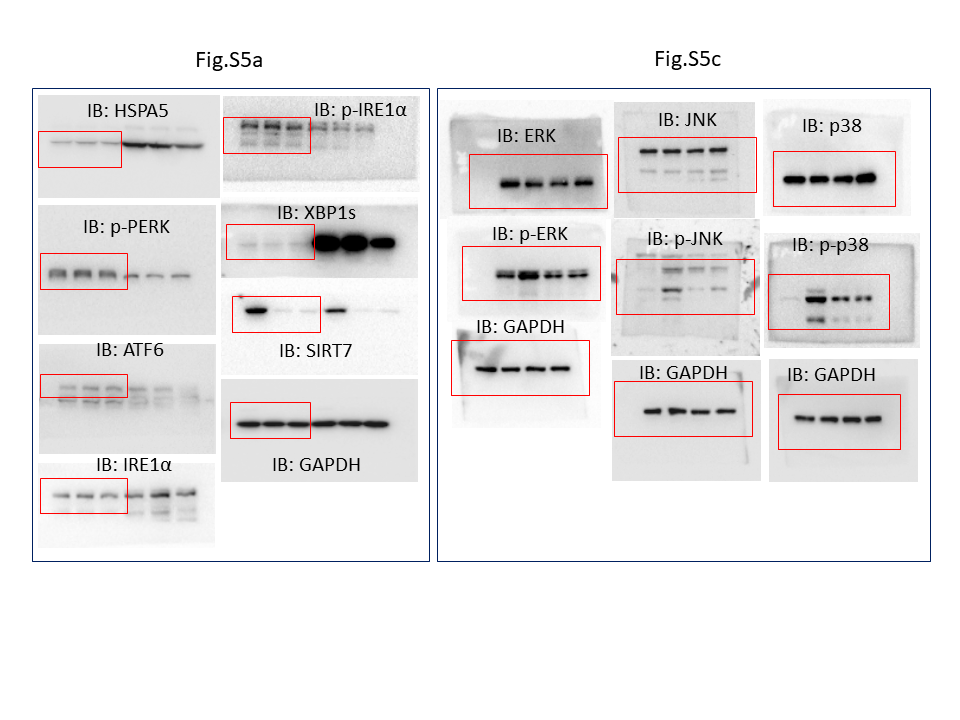


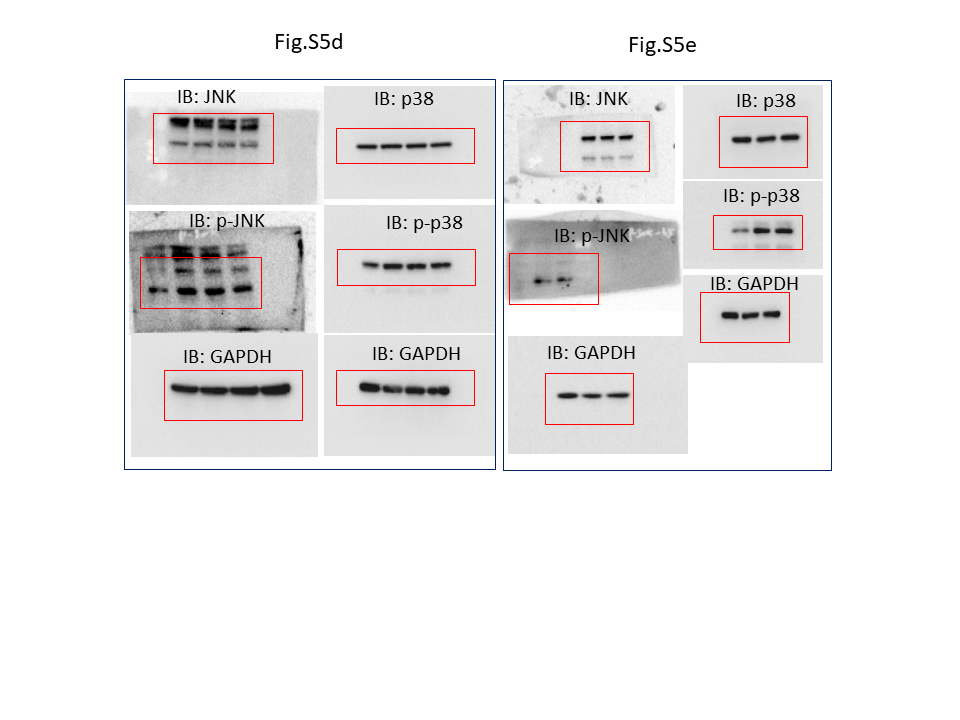


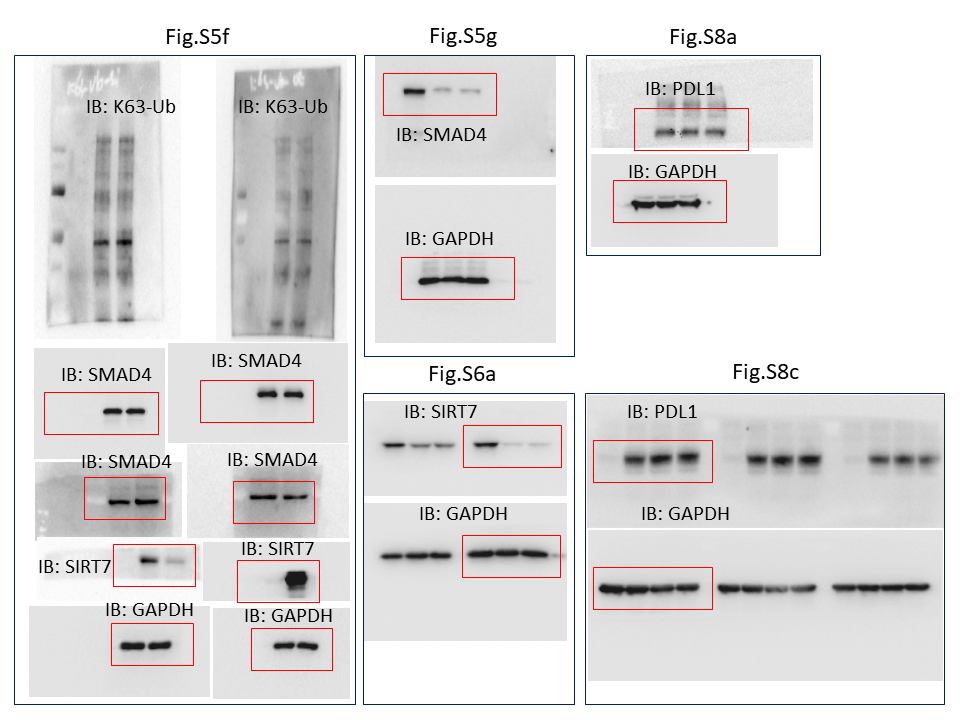


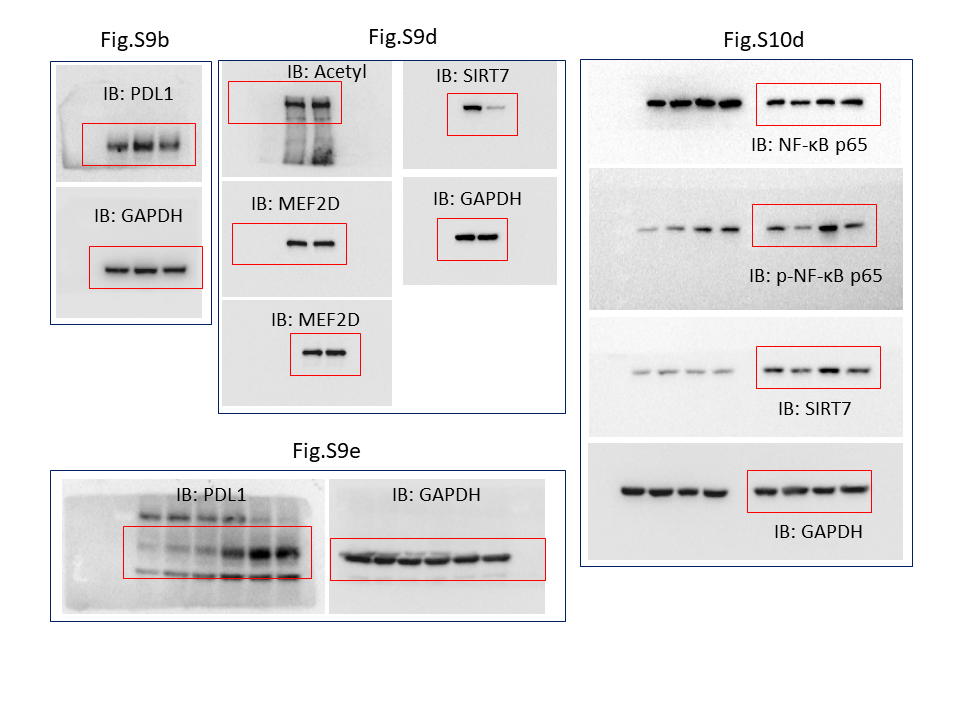

Supplement: Supplementary file 1 — Supplementary Materials [file 41392_2023_1314_MOESM1_ESM.docx]
